# Enhanced inflammation in New Zealand white rabbits when MERS-CoV reinfection occurs in the absence of neutralizing antibody

**DOI:** 10.1371/journal.ppat.1006565

**Published:** 2017-08-17

**Authors:** Katherine V. Houser, Andrew J. Broadbent, Lisa Gretebeck, Leatrice Vogel, Elaine W. Lamirande, Troy Sutton, Kevin W. Bock, Mahnaz Minai, Marlene Orandle, Ian N. Moore, Kanta Subbarao

**Affiliations:** 1 Laboratory of Infectious Diseases, National Institute of Allergy and Infectious Disease, National Institutes of Health, Bethesda, MD, United States of America; 2 Comparative Medicine Branch, Infectious Disease Pathogenesis Section, National Institute of Allergy and Infectious Disease, National Institutes of Health, Bethesda, MD, United States of America; Division of Clinical Research, UNITED STATES

## Abstract

The Middle East respiratory syndrome coronavirus (MERS-CoV) is a zoonotic betacoronavirus that was first detected in humans in 2012 as a cause of severe acute respiratory disease. As of July 28, 2017, there have been 2,040 confirmed cases with 712 reported deaths. While many infections have been fatal, there have also been a large number of mild or asymptomatic cases discovered through monitoring and contact tracing. New Zealand white rabbits are a possible model for asymptomatic infection with MERS-CoV. In order to discover more about non-lethal infections and to learn whether a single infection with MERS-CoV would protect against reinfection, we inoculated rabbits with MERS-CoV and monitored the antibody and inflammatory response. Following intranasal infection, rabbits developed a transient dose-dependent pulmonary infection with moderately high levels of viral RNA, viral antigen, and perivascular inflammation in multiple lung lobes that was not associated with clinical signs. The rabbits developed antibodies against viral proteins that lacked neutralizing activity and the animals were not protected from reinfection. In fact, reinfection resulted in enhanced pulmonary inflammation, without an associated increase in viral RNA titers. Interestingly, passive transfer of serum from previously infected rabbits to naïve rabbits was associated with enhanced inflammation upon infection. We further found this inflammation was accompanied by increased recruitment of complement proteins compared to primary infection. However, reinfection elicited neutralizing antibodies that protected rabbits from subsequent viral challenge. Our data from the rabbit model suggests that people exposed to MERS-CoV who fail to develop a neutralizing antibody response, or persons whose neutralizing antibody titers have waned, may be at risk for severe lung disease on re-exposure to MERS-CoV.

## Author summary

New Zealand white rabbits display an increase in lung inflammation following reinfection with MERS-CoV that is associated with non-neutralizing antibodies and complement proteins. The development of neutralizing antibodies resulted in protection from infection. These findings may have implications for individuals that fail to develop a neutralizing antibody response, or for those whose response wanes over time, upon re-exposure to MERS-CoV.

## Introduction

Since its discovery in 2012, the Middle East respiratory syndrome coronavirus (MERS-CoV) has caused at least 2,040 human infections and 712 deaths worldwide [1, 2]. Like other human coronaviruses (229E, OC43, NL63 and HKU1), MERS-CoV is associated with respiratory tract infection. However, unlike most other human coronaviruses, MERS-CoV has a zoonotic origin and can cause severe illness, resulting in acute respiratory distress syndrome. These characteristics are reminiscent of severe acute respiratory syndrome coronavirus (SARS-CoV), which caused a large outbreak of human infections in 2003 [2].

Serological surveys of persons in the Arabian Peninsula have shown low or undetectable levels of preexisting antibody against MERS-CoV, although those in close contact with camels (the reservoir host for MERS-CoV) have higher rates of seropositivity than the general population [3–5]. Longitudinal studies have also indicated that serum antibody titers may wane over time, particularly following mild infections [6–8]; similar to what has been observed for other coronaviruses like SARS-CoV [9].

Since the discovery of MERS-CoV, only one autopsy report has been published and the course of MERS-CoV infection in humans is still not well understood [10]. This is particularly true for the mild or asymptomatic infections, which comprise a large number of MERS-CoV infections in healthy adults [11–14]. We wished to explore the immune response during non-lethal MERS-CoV infection, and to determine whether such infections would be protective.

Several small animals, including ferrets, hamsters, and mice which are frequently used as animal models for human disease have proven resistant to infection with MERS-CoV [15–18]. The dipeptidyl peptidase 4 (DPP4) protein, which is the cellular receptor for MERS-CoV in these animals differed from human DPP4 at key residues, and therefore did not bind to the MERS-CoV spike protein [18]. Several modified mouse models have been generated to overcome this receptor-mediated restriction including both transduced and transgenic animals expressing human DPP4, and lethal infection models have been established [19–21]. Non-human primates have been successfully infected, with rhesus macaques displaying a mild, transient illness and marmosets demonstrating a more severe and sometimes lethal infection [22–25], although there is some discrepancy in findings from marmosets [26]. Camels and alpacas have also been experimentally infected and exhibit transient viral replication in the upper respiratory tract [27, 28]. However, the expense and care of camels and the ethical concerns surrounding the use of non-human primates limits their widespread utility for research studies.

The New Zealand white rabbit supports productive replication of the MERS-CoV isolate EMC/2012 without associated clinical signs of disease [29]. We sought to characterize the role of antibodies in protection from reinfection following asymptomatic infection. We found that primary infection failed to induce neutralizing antibodies and reinfection was associated with increased pulmonary inflammation. Reinfection elicited neutralizing antibodies that protected rabbits from subsequent infection. Thus, whilst neutralizing antibodies are protective, they may not be elicited or may not last long after mild infection with MERS-CoV and infection in the presence of only non-neutralizing antibodies may be associated with enhanced pulmonary inflammation.

## Results

### MERS-CoV infection results in a transient pulmonary disease

**Fig 1:**
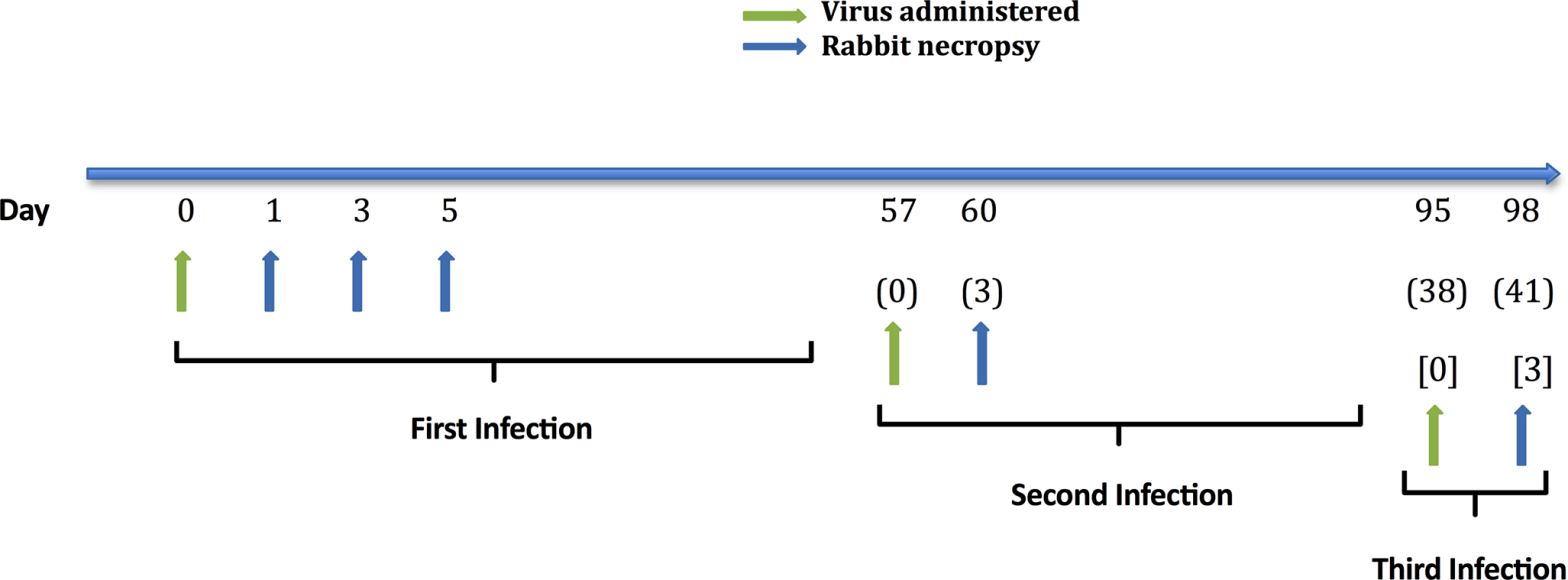
Schematic of rabbit infection studies. Rabbits were inoculated intranasally with EMC/2012 strain of MERS-CoV (green arrows) and tissue samples were collected for viral titration and histopathology at necropsy (blue arrows). Three rabbits were necropsied at each time point. Numbers indicate days since virus administration for primary, (secondary), or [tertiary] infections.

**Fig 2:**
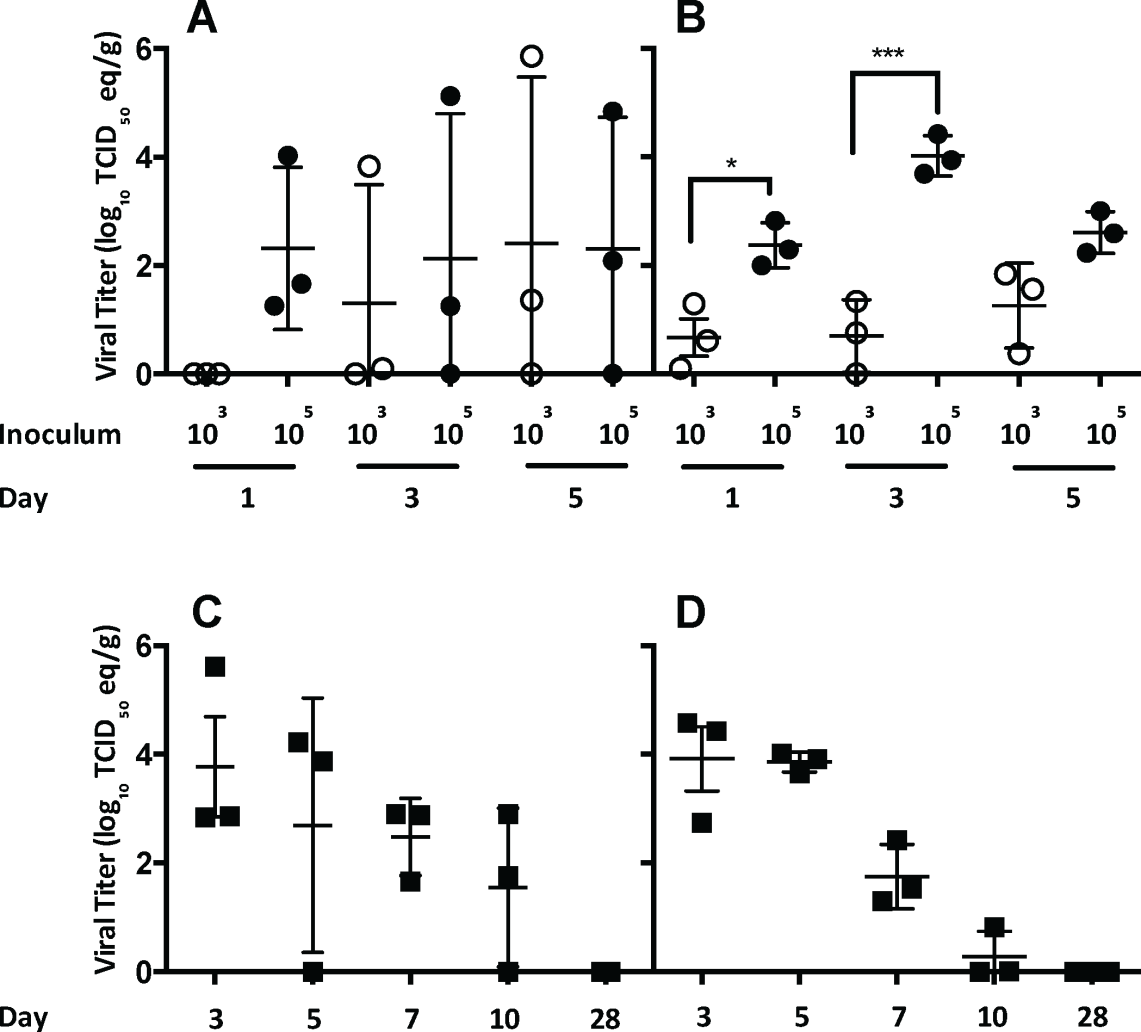
Viral RNA titers in the respiratory tract following primary infection with MERS-CoV. Viral RNA titers in the nasal turbinates (A) and lungs (B) of rabbits following infection with either 10^3^ or 10^5^ TCID_50_ of EMC/2012 strain of MERS-CoV through day 5 after infection. In a separate experiment, viral RNA titers were determined in the nasal turbinates (C) and lungs (D) following infection with 10^6.5^ TCID_50_. n = 3 rabbits per group. Statistical significance was determined using one-way ANOVA with Tukey’s multiple comparisons test. p values *<0.05, ***<0.001.

In order to study the initial disease progression and antibody response associated with MERS-CoV infection in rabbits, we infected nine New Zealand White rabbits with either a low dose (10^3^ TCID_50_) or high dose (10^5^ TCID_50_) of EMC/2012 (Fig 1). None of the rabbits displayed overt clinical signs of illness in the 14 days following infection. Viral RNA titers were measured by qRT-PCR using primer pairs targeting the nucleocapsid protein (N3) and are reported as genome equivalents per gram of tissue [30], as we found this method to be more sensitive and less variable than recovering infectious virus from infected rabbit tissues (Fig 2A and 2B and S1 Fig) as reported previously in the rabbit and other MERS-CoV animal models [22, 29].

We observed a transient infection following inoculation, with detection of viral RNA largely limited to the respiratory tract. In the nasal turbinates, viral RNA was detected only sporadically, although the titers increased after day 1 post-infection. In general, the higher dose of virus resulted in greater mean genome equivalent titers than infection with the lower dose of MERS-CoV (Fig 2A). A dose-response was observed in the lower respiratory tract; with the 10^5^ TCID_50_ inoculum resulting in significantly higher titers than infection with 10^3^ TCID_50_ of virus on days 1 and 3 post-infection (Fig 2B) (p values of 0.03 and 0.0001 respectively).

**Fig 3:**
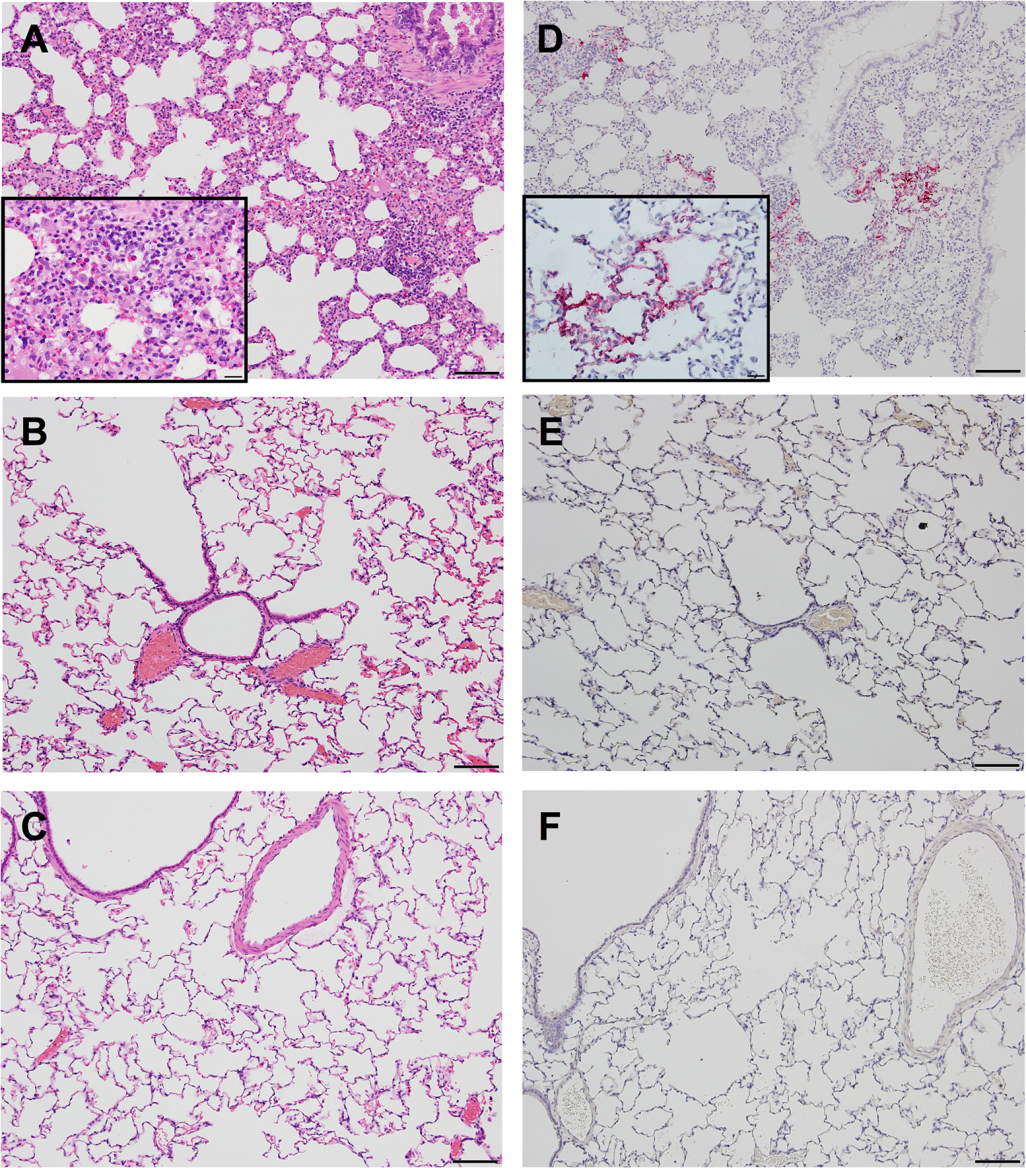
Histopathology in the lungs following primary infection with EMC/2012 strain of MERS-CoV. Images show H&E (left) and IHC against the MERS-CoV N protein (right) following infection with 10^5^ TCID_50_ (A,D), 10^3^ TCID_50_ (B,E), or a media only control (C,F). All images at 10x, (bar equivalent to 100μm) with 40x insets (bar equivalent to 20μm). Images shown are from day 3 post-infection for all groups.

Following primary infection with 10^5^ TCID_50_, mild inflammation involving the perivascular, peribronchiolar, and alveolar interstitial regions was observed in the lungs at day 3 post-infection, with little to no cellular debris within airways (Fig 3A, S1 and S3 Tables). The cellular infiltrate was largely composed of eosinophils and macrophages and fewer lymphocytes and plasma cells (Fig 3A inset). Inflammation was not observed following infection with 10^3^ TCID_50_ of virus or in media-only controls (Fig 3B and 3C and S1 and S3 Tables) based on blind scoring and digital quantitative analysis. Immunohistochemistry (IHC) revealed virus antigen following infection with the higher (10^5^ TCID_50_) dose of virus (Fig 3D and inset, S1 and S2 Tables), but not the lower dose or media-only inoculum (Fig 3E and 3F and S1 and S2 Tables).

In a separate experiment, rabbits were inoculated with 10^6.5^ TCID_50_ of EMC/2012 (Fig 2C and 2D). Viral RNA titers in the lung were sustained until day 5, then dropped to almost baseline levels by day 10, and were undetectable on day 28 post-infection.

Serum was collected for detection of MERS-specific antibodies by both ELISA and microneutralization (MN) assays (Table 1). Eight weeks after inoculation, serum antibodies against the S protein were detected by IgG ELISA in all of the rabbits inoculated with 10^5^ TCID_50_ [geometric mean titer (GMT) 1016], but not in the group inoculated with 10^3^ TCID_50_. Antibodies against the nucleocapsid (N) protein were detected by IgG ELISA in two rabbits previously inoculated with 10^5^ TCID_50_ and one rabbit with 10^3^ TCID_50_. However, neutralizing antibodies were not detected in rabbits inoculated with any dose (10^3^, 10^5^, or 10^6.5^) of EMC/2012 (Table 1).

**Table 1:** Serum ELISA and neutralizing antibody titers in rabbits following primary and secondary infection.

Infection	Inoculum dose^a^	Time post primary infection (weeks)	S ELISA titers GMT^b^ (# with detectable antibody)^c^	N ELISA titers GMT (# with detectable antibody)	MN titers GMT (# with detectable antibody)
Primary	10^3^	8	<10^d^ (0)	100 (1)	<10 (0)
Primary	10^5^	8	1016 (3)	158 (2)	<10 (0)
Secondary	10^3//^10^5^	13	6451 (3)	635 (3)	27 (2)
Secondary	10^5//^10^5^	13	4064 (3)	400 (1)	73 (3)
Passive transfer	10^3^	4	10 (1)	ND^e^	<10 (0)

^a^ // indicates the sequence of subsequent infections

^b^ GMT- geometric mean titer

^c^ # with detectable antibody titer out of 3 rabbits

^d^ <10 indicates titers were below the limit of detection

^e^ ND- not determined

These data indicate that there is a dose-response in MERS-CoV infected rabbits measured by viral RNA and antibody titers. The peak in viral titers occurs at day 3 post-infection, with higher titers observed following infection with 10^5^ or 10^6.5^ TCID_50_ of virus. Since the 10^5^ and 10^6.5^ TCID_50_ doses gave similar results, the 10^5^ TCID_50_ dose was chosen for the remaining studies.

### Neither anti-S nor anti-N protein antibodies provide protection from reinfection

Our findings in rabbits are reminiscent of a few reports of human cases of qRT-PCR confirmed infection with MERS-CoV that failed to elicit either a neutralizing antibody response, or any detectable antibody response against the virus [6, 8, 31]. In order to determine whether such patients would be susceptible to reinfection, we repeated the MERS-CoV infection in the previously infected rabbits. Eight weeks after primary infection, we challenged six rabbits that had previously received the high or low dose of MERS-CoV with 10^5^ TCID_50_ of EMC/2012 (Fig 1). Additional naïve rabbits were inoculated for comparison. As in primary infection, clinical signs were not observed upon reinfection.

**Fig 4:**
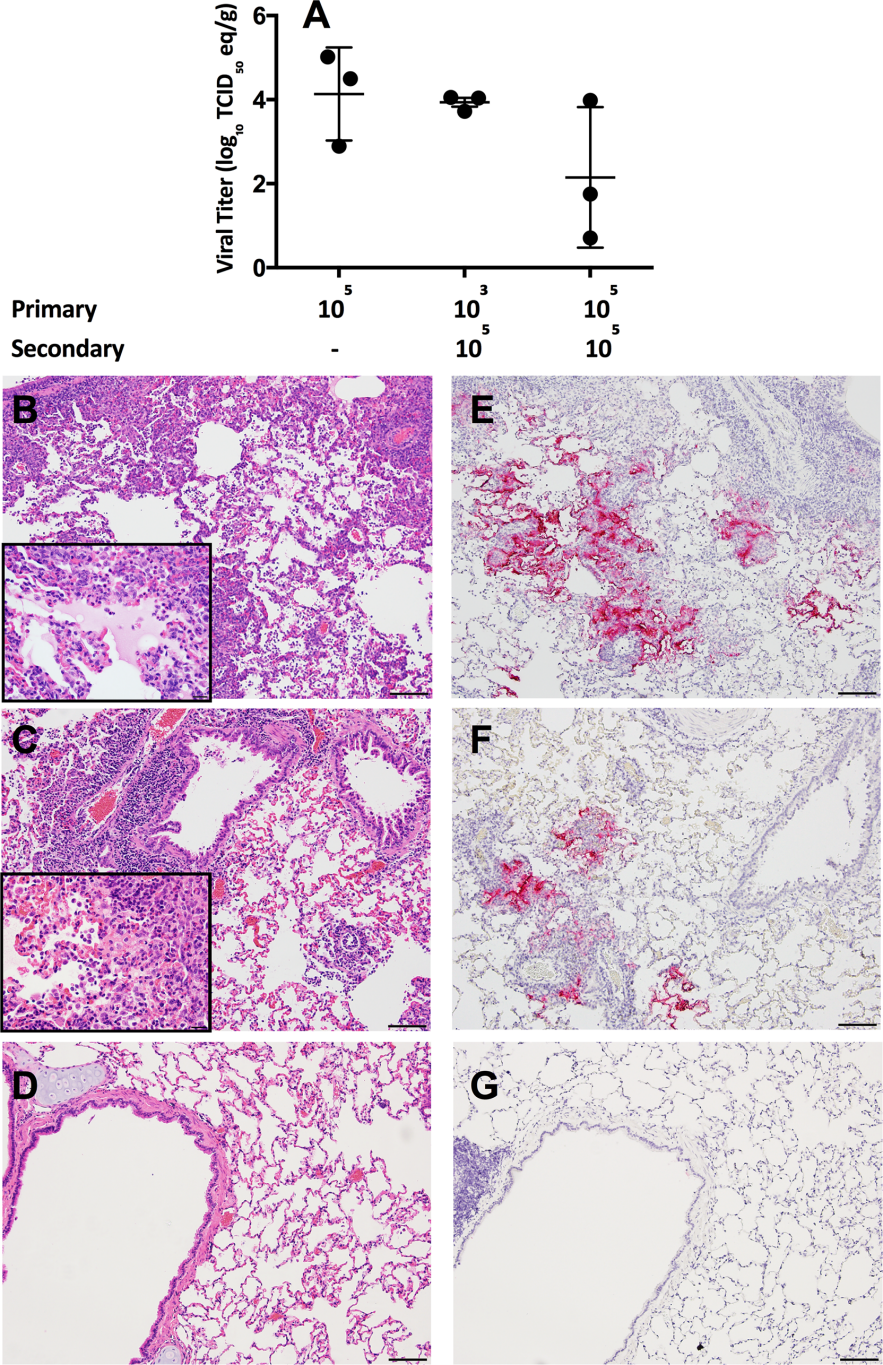
Viral RNA titers and histopathology in the lungs of rabbits following reinfection with MERS-CoV. Viral RNA titers in the lungs of rabbits following reinfection with EMC/2012 (A). Images show H&E (left) and IHC for the MERS-CoV N protein (right) following reinfection for the 10^3//^10^5^ TCID_50_ reinfection group (B,E) and 10^5//^10^5^ TCID_50_ reinfection group (C,F). The 10^5^ TCID_50_^//^media control group was included to demonstrate that the observed inflammation was not residual from the primary infection (D,G). n = 3 rabbits per group. All images at 10x, (bar equivalent to 100μm) with 40x inset (bar equivalent to 20μm). Images from day 3 post-infection.

Neither group of reinfected rabbits had viral RNA detected in the upper respiratory tract, although viral RNA was detected in the primary infection control group (S2 Fig). However, all groups had evidence of pulmonary infection. The rabbits infected serially with 10^5^ TCID_50_ of EMC/2012 (10^5//^10^5^) had lower viral RNA titers compared to both the 10^3//^10^5^ TCID_50_ and 10^5^ TCID_50_ primary infection groups, with mean titers of 10^2.9^, 10^3.7^, and 10^4^ TCID_50_ eq per gram of tissue respectively (Fig 4A).

Inflammatory changes were more severe upon reinfection compared to primary infection, with the greatest inflammation observed in the animals previously infected with the low dose of virus (Fig 3A, 4B and 4C, and S1 and S3 Tables). These severely inflamed regions were characterized by an abundance of eosinophils, macrophages, lymphocytes and plasma cells which formed densely cellular collars of inflammatory cells around the affected perivascular and peribronchiolar regions. In addition, the cellular infiltrate expanded and obscured much of the adjacent alveolar interstitium. The alveolar interstitium also contained regions of proteinaceous fluid and diffuse type II pneumocyte hyperplasia (Fig 4B inset). This inflammatory response was driven by reinfection, and was not residual inflammation from the primary infection. This was confirmed by including a group of previously infected rabbits that received diluent alone in the reinfection study (Fig 4D, and S1 Table). The rabbits in the 10^3//^10^5^ TCID_50_ group had antigen levels comparable to primary infection, while the 10^5//^10^5^ TCID_50_ group had lower levels of antigen by IHC (Fig 4E and 4F, S1 and S2 Tables). These data indicate that low titers of non-neutralizing antibodies do not protect rabbits from reinfection, and may instead result in enhanced inflammation.

The S protein-specific IgG ELISA antibody titers were boosted following secondary infection and remained detectable for five weeks, with a GMT of 6451 for the 10^3//^10^5^ TCID_50_ group, and a GMT of 4064 for the 10^5//^10^5^ TCID_50_ group (Table 1). N protein-specific antibodies were found in all rabbits in the 10^3//^10^5^ TCID_50_ group and one of the 10^5//^10^5^ TCID_50_ group. Secondary infections in both groups resulted in the production of neutralizing antibodies, although the titer in one rabbit in the 10^3//^10^5^ TCID_50_ group dropped below the detection limit by five weeks post-infection (Table 1). Overall the 10^3//^10^5^ TCID_50_ group had lower neutralizing titers than the 10^5//^10^5^ TCID_50_ group, with GMTs of 27 and 73 respectively.

### Neutralizing antibodies provide protection from infection

**Fig 5:**
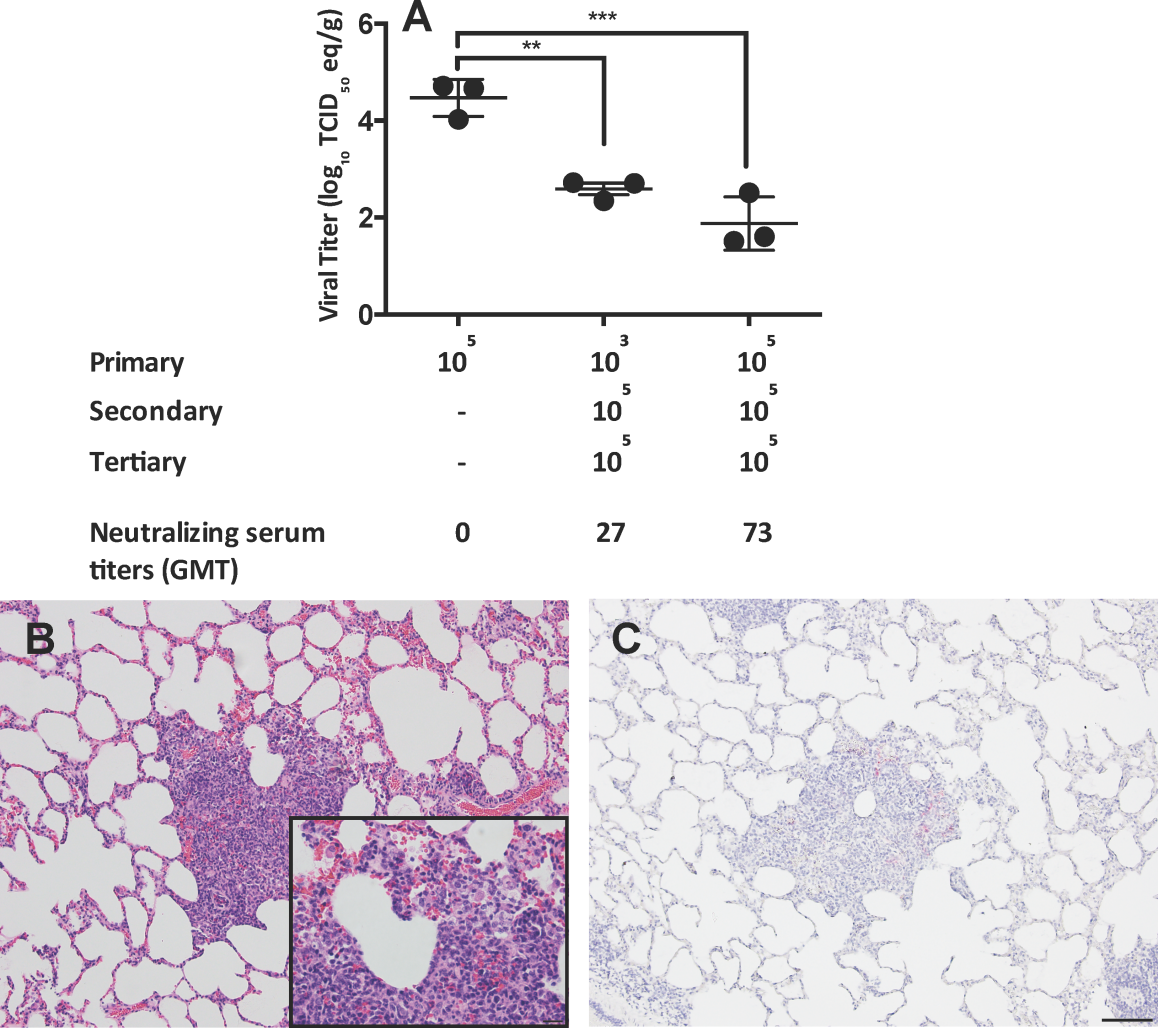
Viral RNA titers and histopathology in lungs following infection with MERS-CoV when neutralizing antibodies are present. Viral RNA titers in the lungs following tertiary infection with EMC/2012 strain (A). Images show H&E staining (B) and IHC with an antibody against the MERS-CoV N protein (C) in the 10^5//^10^5//^10^5^ TCID_50_ group. Images are representative of all rabbits following tertiary infection. All images at 10x, (bar equivalent to 100μm) with 40x inset (bar equivalent to 20μm). n = 3 rabbits per group. Statistical significance was determined using one-way ANOVA with Tukey’s multiple comparisons test. Images from day 3 post-infection. p values **<0.01, ***<0.001.

To determine if neutralizing antibodies would protect from reinfection, three rabbits from each secondary infection group were re-challenged with 10^5^ TCID_50_ EMC/2012 five weeks later (Fig 1). Clinical signs of illness were not observed in any of the rabbits. As was observed with the second infection, viral RNA was not detected in the upper respiratory tract samples from either group of rabbits on day 3 post-infection. In the lungs, a significant decrease in the amount of viral RNA was observed in both the 10^5//^10^5//^10^5^ TCID_50_ group and the 10^3//^10^5//^10^5^ TCID_50_ group compared to primary infection, with mean titers of 10^1.9^, 10^2.6^, and 10^4.5^ TCID_50_ eq per gram of tissue respectively (p values of 0.0006 and 0.003) (Fig 5A). This decrease in viral load was also observed by IHC (S1 and S2 Tables).

Histologically, the lungs from both groups displayed mild inflammation and minimal antigen burden (Fig 5B and 5C and S1, S2 and S3 Tables). In these milder regions of peribronchiolar and perivascular inflammation, eosinophils and macrophages predominated (Fig 5B inset).

Thus, infection in the presence of neutralizing antibodies was associated with significant protection from viral infection and associated pathology in both the upper and lower respiratory tract of the rabbits. Moreover, the prechallenge serum neutralizing antibody titers inversely correlated with viral RNA titers following tertiary infection (Fig 5A).

### Non-neutralizing antibodies mediate enhanced inflammation following reinfection

**Fig 6:**
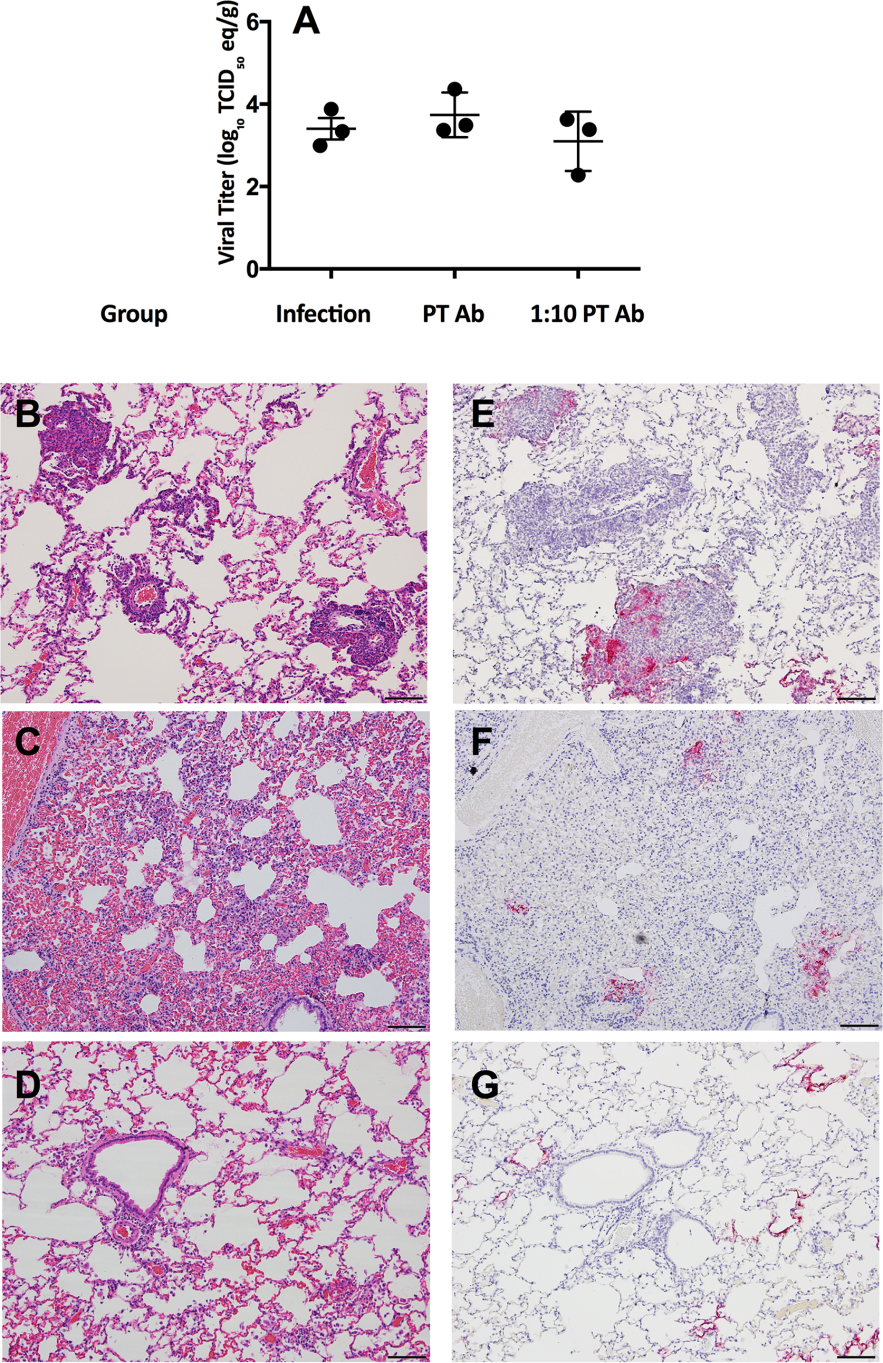
Viral RNA titers and histopathology in the lungs following MERS-CoV infection in rabbits that received passive transfer (PT) of serum from infected rabbits. Viral RNA titers in the lungs upon infection with 10^5^ TCID_50_ of MERS-CoV in rabbits either previously infected with a low dose of MERS-CoV (10^3^ TCID_50_) four weeks prior or naïve rabbits following PT of post-infection sera at either a full dose or 1:10 dilution (A). Images show the H&E staining (left) and IHC with an antibody against the MERS-CoV N protein (right) following infection for the 10^3//^10^5^ TCID_50_ (reinfection) group (B,E), the group that received passive transfer of undiluted post-infection serum (C, F), and the group that received passive transfer of post-infection serum at 1:10 dilution (D,G). n = 3 rabbits per group. Images from day 3 post-infection at 10x, bar equivalent to 100μm.

To determine if non-neutralizing antibodies were responsible for the enhanced inflammation observed following reinfection, we performed a passive transfer (PT) experiment. Serum collected from rabbits four weeks following primary infection with 10^3^ TCID_50_ of EMC/2012 was transferred either undiluted or at a 1:10 dilution in PBS to naïve rabbits that were challenged with 10^5^ TCID_50_ of virus the following day. For comparison, a group of previously infected rabbits were reinfected. Although ELISA antibodies against the S protein were barely detectable in the serum (Table 1), after the serum was concentrated ten-fold prior to administration the ELISA titers ranged from 10 to 40. Neutralizing activity was not detected, even after concentration of the serum. Thus, very low titers of non-neutralizing antibodies were present in the transferred serum. Passively transferred antibodies did not affect viral titers in the lower respiratory tract as determined by qRT-PCR (Fig 6A).

Rabbits that received the undiluted passively transferred (PT) serum exhibited immunopathology similar to that observed in previously infected rabbits based on blinded scoring (Fig 6B and 6C and S1 Table). There was an increase in observed vascular congestion in this group of PT rabbits compared to the other groups. The rabbits that received serum antibodies at the lower dilution did not demonstrate enhanced inflammation (Fig 6D, S1 Table). Overall, the pathology in the rabbits that were infected after PT of post-infection serum was milder than in other reinfection studies, possibly due to the shortened interval between primary infection and reinfection. Viral antigen levels appeared similar between all groups by IHC (Fig 6E, 6F and 6G, S1 and S2 Tables).

### Increased complement activation is associated with enhanced inflammation

Non-neutralizing antibodies typically enhance inflammation and pathology during an immune response through interactions with Fc or complement receptors [32]. We first examined the possibility that the antibodies were causing enhanced inflammation due to an increase in viral uptake and replication in macrophages through interaction with their native cellular receptor or an Fc receptor, as happens in dengue [32–34]. Antibody-dependent enhancement (ADE) during infection has also been observed with other coronaviruses, such as feline infectious peritonitis virus (FIPV) [35]. However, it was not clear that such a mechanism was likely since the enhanced inflammation in secondary MERS-CoV infection in rabbits was not associated with an increased viral load by either qRT-PCR or IHC (Fig 4A, S1 and S2 Tables).

**Fig 7:**
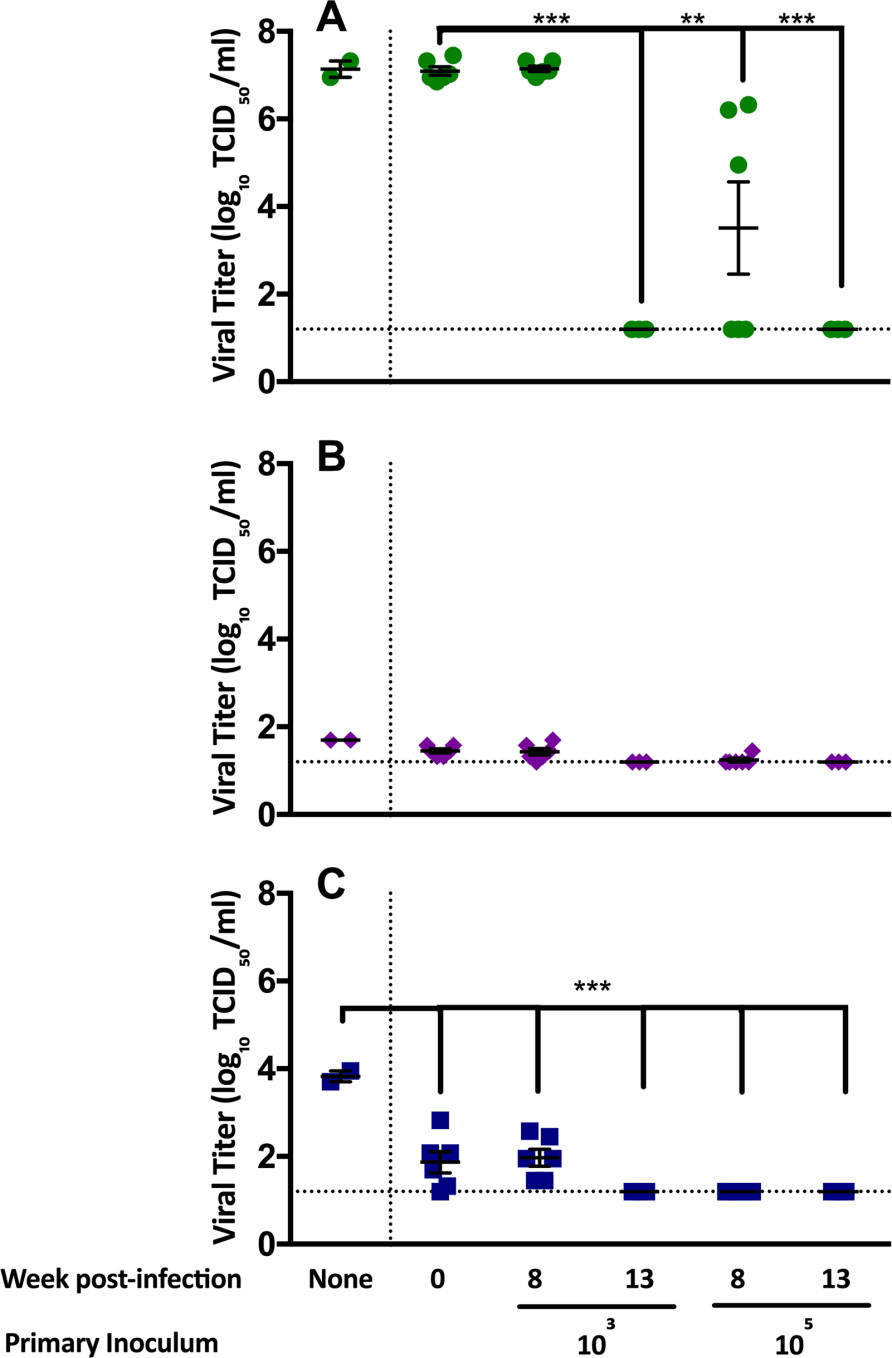
Antibody-dependent enhancement (ADE) assay using rabbit sera throughout the infection series. Sera from naïve rabbits (week 0), following primary infection (week 8), and following secondary infection (week 13) were collected from both the 10^3^ and 10^5^ infection schedules. Week 8 serum had no neutralizing activity while week 13 serum had neutralizing activity. Sera were tested in Vero81 cells (A), Raji cells (B) and THP-1 cells (C). None = virus only control. p values **<0.01, ***<0.001.

In order to examine the replication of MERS-CoV in macrophages, we differentiated THP-1 cells into macrophages and infected them with MERS-CoV in the presence or absence of rabbit sera and measured viral titers after 48 hours. Heat-inactivated sera from naïve rabbits (week 0), week 8 following primary infection (only non-neutralizing antibodies present), and week 13 following secondary infection (neutralizing antibodies present) were tested at three dilutions. All dilutions displayed similar trends but only undiluted samples are shown in Fig 7. Vero81 cells were used as a positive control (Fig 7A) and Raji cells were included as a negative control (Fig 7B) of infection. Compared to the level of viral replication observed in macrophages without serum, addition of rabbit sera produced no enhancement of viral replication. In fact, in the presence of rabbit sera, there was a significant decrease in viral replication in THP-1 cells compared to titers in the absence of rabbit serum (Fig 7C), indicating that the non-neutralizing antibodies did not enhance MERS-CoV replication in these cells.

**Fig 8:**
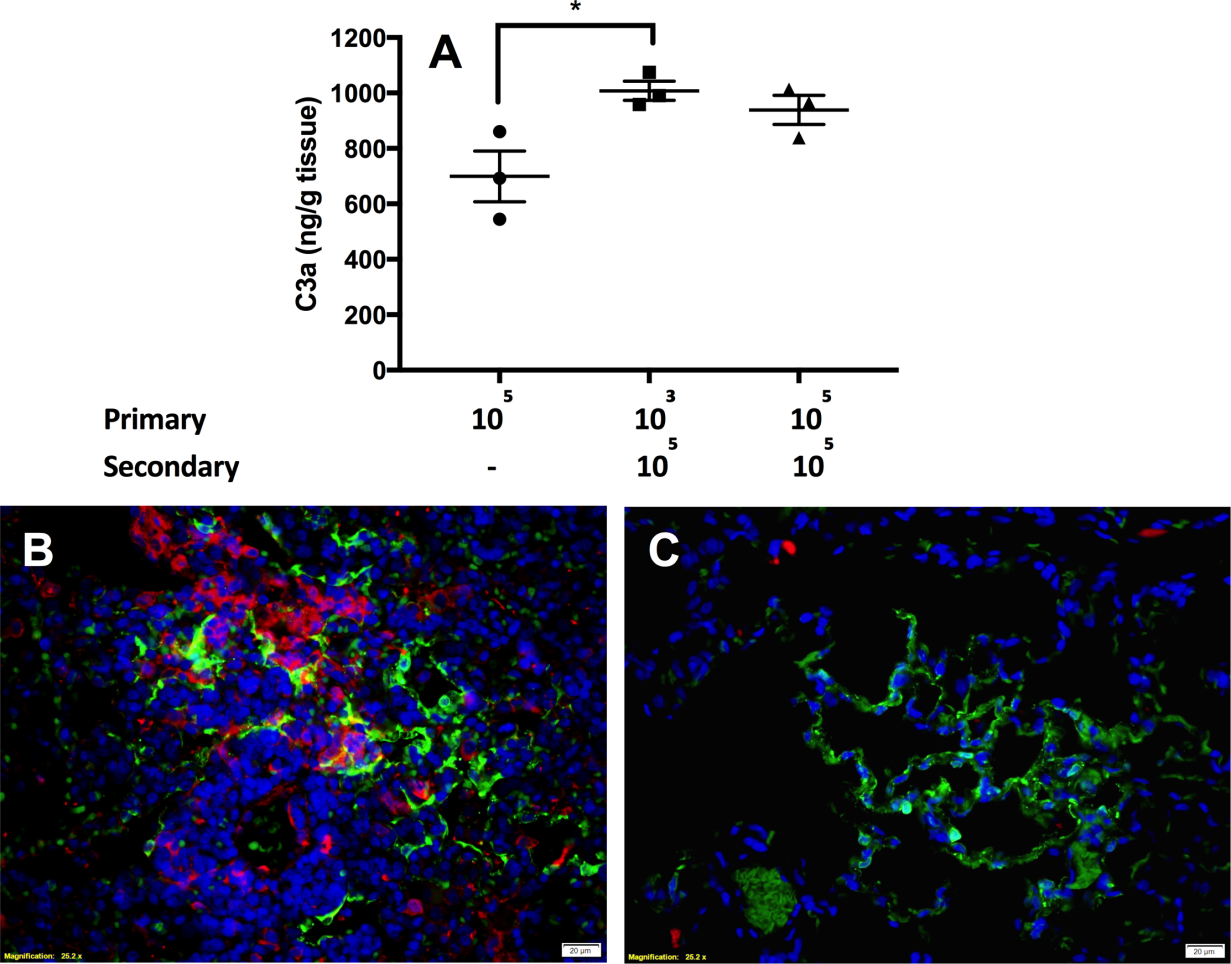
Detection of complement protein during primary infection and reinfection. ELISA against C3a protein in rabbit lung homogenates (A) show an increase in C3a levels present during reinfection compared to primary infection. Immunofluorescence images show MERS-CoV N antigen (green) and complement (red) following secondary infection (B) and primary infection (C). Images from day 3 post-infection at 40x, bar equivalent to 20μm. n = 3 rabbits per group. Statistical significance was determined using one-way ANOVA with Dunnett’s multiple comparisons test. p values *<0.05.

The other possibility for ADE of inflammation is through interaction with complement receptors. We investigated the potential role of complement in the enhanced pulmonary inflammation by evaluating lung samples from primary and secondary MERS-CoV infections with an ELISA against rabbit complement protein C3a. Using this assay, we observed an increased amount of complement protein per gram of lung tissue in both secondary infection groups (mean of 1084 ng/g for 10^3//^10^5^ and 939 ng/g for the 10^5//^10^5^) compared to primary infection (mean value of 699 ng/g) (Fig 8A). This increase was significant for the 10^3//^10^5^ group compared to primary infection (p = 0.02).

We further validated the association between complement and increased inflammation using an anti-complement (C9) antibody. Immunofluorescence revealed complement recruitment through the deposition of virus antigen and C9 within the inflammatory milieu surrounding many vessels and airways in the lungs of the reinfected rabbits (Fig 8B). This was in direct contrast to the primary infection group in which virus antigen was detected adjacent to small vessels and airways with minimal inflammation and no evidence of complement deposition (Fig 8C). Staining for other complement targets (C1q, C4b, C3a, and C3c) was unsuccessful in the rabbit tissues.

### An increase in CD3+ cells occurs during reinfection in the lungs

**Fig 9:**
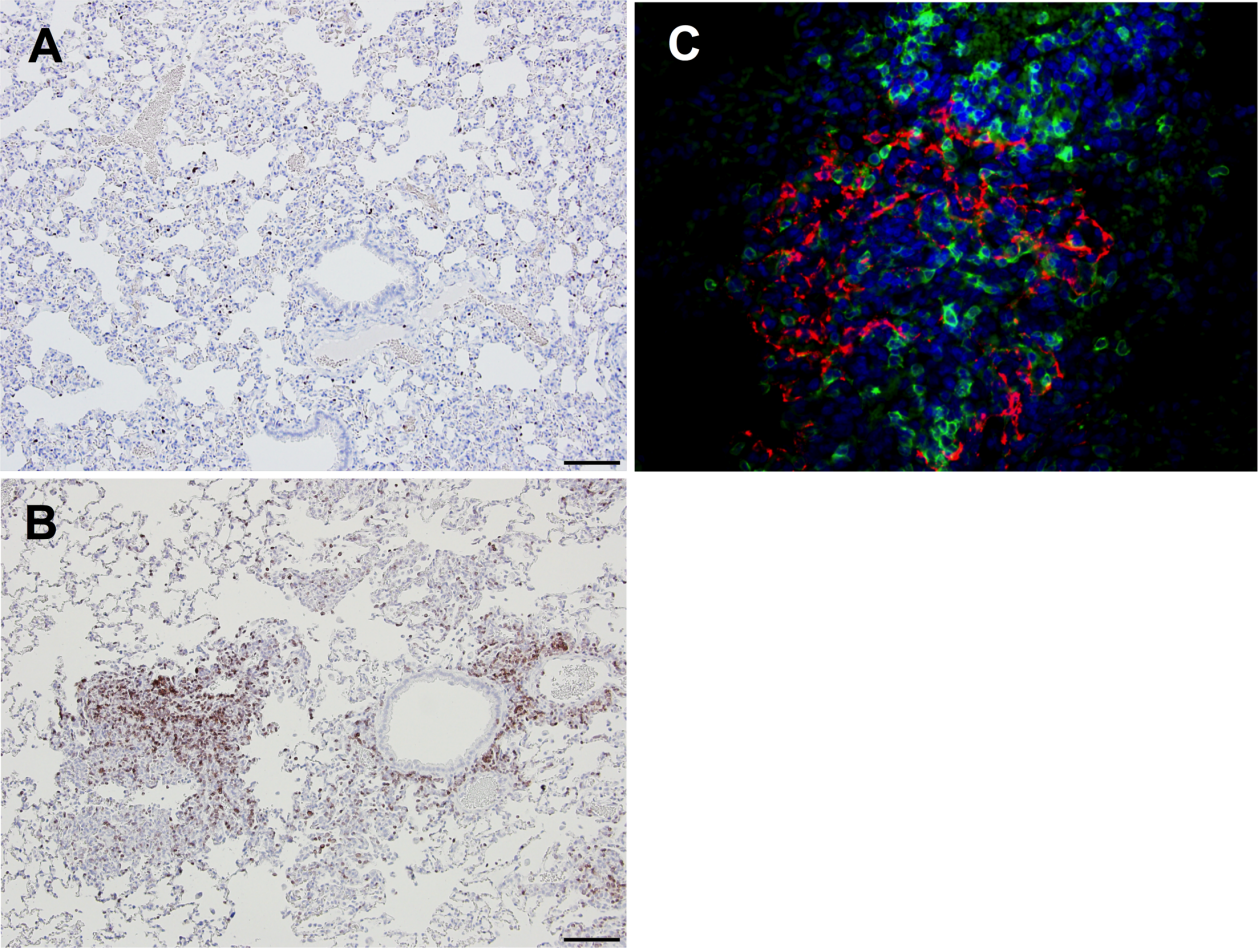
CD3+ cells in the lungs following primary infection and reinfection. DAB images from primary infection (A) and reinfection (B). Immunofluorescence (IF) image of CD3 (green) and virus antigen (red) within the same perivascular region following reinfection (C). DAB images from day 3 post-infection at 10x, bar equivalent to 100μm. IF images at 40x, bar equivalent to 20μm.

Since T cell responses could also be involved in enhanced inflammation, we stained lung tissues with an anti-CD3 antibody (Fig 9). Following reinfection, we observed a substantial increase in the numbers of CD3+ T cells in the lung compared to primary infection (Fig 9A and 9B). These T cells were distributed in the same areas as virus antigen, largely in the areas immediately surrounding vessels and airways (Fig 9C). Attempts to further characterize the CD3+ cells were unsuccessful.

## Discussion

While MERS-CoV is able to cause severe disease with a lethal outcome, many otherwise healthy individuals display a mild or asymptomatic disease course. Using the rabbit model, we examined the serum response following asymptomatic infection, and found that an antibody response lacking neutralizing activity was not protective against reinfection. Our study extends previously published information on the rabbit model [29] by examining a dose response and exploring the enhanced inflammation observed during reinfection. The inclusion of a low dose of virus in our studies revealed the potentially detrimental effects of non-neutralizing antibodies and further demonstrated the protective benefit of neutralizing antibodies [21, 36, 37]. To our knowledge, the rabbit infection model described here is the only model of MERS-CoV infection in which non-neutralizing antibodies are exclusively elicited following primary infection.

Our observations in the rabbit are in general agreement with those reported by Haagmans et al [29], with the exception that neutralizing antibodies were not detected following primary infection in our study. This is likely a consequence of different routes of inoculation. Haagmans and colleagues inoculated rabbits through both the intranasal and intratracheal routes, whereas in our study rabbits were inoculated through the intranasal route alone. In addition, the volume of the inoculum was not stated by Haagmans et al, although a recent publication indicates a volume up to 3ml may have been delivered intratracheally [38]. This differs significantly from the volume used in our studies (1ml) and may affect viral load. Differences in inoculation routes and volumes have been shown to affect disease severity and immune response in other models [39, 40]. The lack of neutralizing antibodies in the rabbits in our model allows us to examine a phenomenon that is otherwise only observed in humans. The potential clinical implication of our findings is a risk of severe pulmonary disease in persons who fail to develop a neutralizing antibody response following exposure to MERS-CoV or in persons in whom titers of neutralizing antibodies decay and are no longer detectable.

In rabbits, the highest mean viral RNA levels were observed in the lower respiratory tract, as has been reported in human cases [10, 41–43]. A direct correlation was observed between the distribution and amount of viral antigen and inflammation in the lungs of the rabbits. In a limited number of samples, aberrantly high levels of viral antigen were observed by both qualitative and quantitative measures that were not mirrored in the qRT-PCR results (S1 and S2 Tables). We believe this occurred due to sampling of locations where the inoculum pooled after infection, particularly since these atypical values occurred most often in the caudal lobes.

The cellular infiltrates observed in the rabbit lungs were largely composed of mixed populations of eosinophils and histiocytes. In mammalian species, the presence of eosinophilic inflammation is often associated with parasitic infections, hypersensitivity reactions, and less often, certain fungal infections [44]. There was no evidence of parasitic or fungal infection in the rabbits and the commonly observed features of hypersensitivity-related pneumonitis (i.e. edema and bronchiolitis) were absent [45].

Viral replication in the upper respiratory tract was detected only during primary infection, suggesting that immune responses prevented local replication of the virus during later challenge. While we only analyzed serum IgG antibodies by ELISA, mucosal IgA antibodies in the upper respiratory tract may also play a role in preventing reinfection. However, the serum IgG antibody lacked neutralizing activity and contributed to the enhanced pulmonary inflammation observed upon reinfection. Passive transfer of serum from previously infected rabbits to naïve animals followed by MERS-CoV challenge recreated the same histopathological depiction, though there was an increase in vascular congestion following passive transfer that was not observed in the previously infected rabbits. This is likely a consequence of transferring complete serum containing additional serum proteins, including those involved in the complement cascade into naïve rabbits instead of transferring purified MERS-CoV specific IgG antibodies. As we observed in these studies, the additional complement proteins could have led to increased inflammation and congestion in the lungs.

Even in the presence of the enhanced inflammation following reinfection, the rabbits continued to lack any discernible clinical signs of infection. This occurrence could be explained by several factors. Rabbits are prey animals, which have evolved to mask signs of illness as a defense mechanism. Also, with the experiments being conducted in a high containment facility, we were limited in our ability to measure activity levels. Furthermore, the lesions in the lungs were typically multifocal and focally severe. The remaining lung tissue may have been sufficiently functional to limit clinical signs.

The effect of non-neutralizing antibodies observed in this MERS-CoV study differs from those observed with FIPV and flaviviruses such as dengue, since the non-neutralizing antibodies did not enhance MERS-CoV replication. The enhanced inflammation observed in the rabbits after MERS-CoV reinfection appears to be mediated through interactions between non-neutralizing antibodies and complement proteins, resulting in activation of the complement cascade and formation of immune complexes. The increase of C3a and C9 proteins in the rabbit lungs following reinfection supports this possibility. Immune complexes have been implicated in the pathogenesis in other viral infections, including influenza and RSV [46, 47]. While C9 and the formation of membrane attack complexes (MACs) are typically involved in response to bacterial pathogens, enveloped viruses are also susceptible to lysis by MACs [48, 49]. Complement activation can also be responsible for an increase in the release of anaphylotoxins and more recruitment and activation of immune cells, leading to inflammation. The presence of CD3+ T cells in the same regions of the lung as MERS-CoV N antigen during reinfection is consistent with this scenario (Fig 9).

Our studies demonstrated that MERS-CoV reinfection elicited neutralizing antibodies that protected rabbits from further viral challenge. Antibody-mediated protection has also been exhibited in rabbits and mice following prophylaxis with neutralizing monoclonal antibodies against the MERS-CoV spike protein [21, 36, 37]. These data support the induction of neutralizing antibodies as the primary goal for vaccines. However, the use of convalescent serum for treatment of MERS-CoV infected individuals has had limited, if any, benefit [50–52]. Also, past experience with SARS-CoV triggers a cautionary note. In mouse and hamster models, vaccine-induced neutralizing antibodies prevented or reduced replication of SARS-CoV [53, 54]. In contrast, in ferrets and nonhuman primate models, SARS-CoV antibodies restricted replication of challenge virus but did not prevent pulmonary inflammation [55, 56].

In addition, antibody-dependent enhancement and pulmonary immunopathology was seen following challenge with some vaccine strategies for SARS-CoV, including virus-like particles and inactivated vaccines [57, 58]. There are some data to suggest that MERS-CoV vaccine approaches may also result in immunopathology; as eosinophilic infiltration with enhanced lung pathology was observed in vaccinated transgenic mice following MERS-CoV challenge [59]. Since the mice had neutralizing antibodies before challenge, and had significant reduction in viral titers following challenge compared to control mice, we believe the mechanisms behind these two phenomena are distinct, but still require consideration. These discrepant observations highlight the critical need for additional clinical data, and continued attention during the development and testing of coronavirus vaccines.

Another approach to viewing our data is to consider primary infection in rabbits as a type of vaccination, resulting in an immune response without overt clinical symptoms. This “priming” infection produces an immune response that is inadequate for protection. The secondary infection then acts as a “booster”, activating the memory response elicited by the primary infection and inducing neutralizing antibodies. Either interpretation indicates that the production of neutralizing antibodies should be the goal of MERS-CoV vaccines. Additional vaccine doses may be needed if neutralizing antibody titers wane rapidly.

Rare cases of qRT-PCR confirmed human MERS-CoV infections have been reported in which neutralizing or S protein ELISA antibody responses were not detected [6, 8, 31], most often following mild or asymptomatic infection. The rabbit model, particularly with use of lower viral inoculum dose and volume, may recapitulate such cases. If neutralizing antibodies against MERS-CoV are not produced or wane over time, a mild or asymptomatic infection may prime individuals for more severe disease upon re-exposure. This possibility could occur after either infection or vaccination, and should be considered during the development of MERS-CoV vaccines.

## Materials and methods

### Virus and cells

Vero81 cells (ATCC) were grown and maintained in Opti-MEM media (GIBCO) with 5% FBS (HyClone). Raji (ATCC) and THP-1 cells (ATCC) were maintained in RPMI-1640 media (GIBCO) with 10% FBS and 50μM β-mercaptoethanol (Sigma). The virus HCoV-EMC/2012 was obtained from Erasmus Medical Center, Netherlands. Virus stocks were stored at -80°C. The titer of the stock virus was determined by serial dilution in Vero81 cells and calculated by the Reed and Muench method [60]. All experiments were performed in a biosafety level 3 (BSL3) facility.

### Rabbit infection studies

Male New Zealand white rabbits (Covance, Princeton, NJ) between five to nine months of age were anesthetized with a combination of intramuscular dexmedetomidine and isoflurane inhalation. Animals were inoculated intranasally (i.n.) with virus diluted in 1ml of MERS-CoV in Leibovitz-15 (L15) media (GIBCO), or mock-infected with 1ml of media alone. Atipamezole was subsequently administered subcutaneously to reverse sedation. Rabbits were monitored daily for 14 days after infection for clinical signs of disease including temperature, weight, lethargy, ocular discharge, rhinitis, labored breathing, ruffled fur, inappetence, and diarrhea. Serum was collected via the ear vein prior to inoculation and at specified times following infection. Animals were euthanized by Beuthanasia D administration and tissues were collected for viral titration, histopathology, and immunohistochemistry (IHC). For passive transfer (PT) studies, serum from rabbits infected 28 days prior was concentrated 10-fold using an Amicon Ultra-15 filter column and then transferred intravenously through the ear vein to naïve rabbits either undiluted or at a 1:10 dilution, one day prior to infection with 10^5^ TCID_50_ of MERS-CoV in 1ml. All infections consisted of the EMC/2012 strain unless otherwise noted. All animal studies were conducted in ABSL3 laboratories at the National Institutes of Health (NIH).

### Ethics statement

All procedures were reviewed and approved by the NIAID DIR Animal Care and Use Committee. The animals were housed in rabbit/ferret bio-containment racks and maintained in accordance with the Animal Welfare Act, the Guide for the Care and Use of Laboratory Animals, and other Federal statutes and regulations relating to animals, in a fully AAALAC accredited facility. All procedures were performed utilizing appropriate anesthetics as listed in the NIAID DIR Animal Care and Use Committee approved animal study proposal LID 33E. Euthanasia methods were consistent with the AVMA Guidelines on Euthanasia and the endpoint criteria listed in the NIAID DIR Animal Care and Use Committee approved animal study proposal LID 33E.

The NIAID DIR Animal Care and Use Program, as part of the NIH Intramural Research Program (IRP), complies with all applicable provisions of the Animal Welfare Act (http://www.aphis.usda.gov/animal_welfare/downloads/awa/awa.pdf) and other Federal statutes and regulations relating to animals. The NIAID DIR Animal Care and Use Program is guided by the "U.S. Government Principles for the Utilization and Care of Vertebrate Animals Used in Testing, Research, and Training" (http://oacu.od.nih.gov/regs/USGovtPrncpl.htm).

The NIAID DIR Animal Care and Use Program acknowledges and accepts responsibility for the care and use of animals involved in activities covered by the NIH IRP’s PHS Assurance #A4149-01, last issued 11/24/2014. As partial fulfillment of this responsibility, the NIAID DIR Animal Care and Use Program ensures that all individuals involved in the care and use of laboratory animals understand their individual and collective responsibilities for compliance with that Assurance, as well as all other applicable laws and regulations pertaining to animal care and use.

The NIAID DIR Animal Care and Use Program has established and will maintain a program for activities involving animals in accordance with the most recent (2011, 8^th^ edition) of “The Guide for the Care and Use of Laboratory Animals” (ILAR, NRC) (http://oacu.od.nih.gov/regs/guide/guide_2011.pdf).

The policies, procedures and guidelines for the NIH IRP are explicitly detailed in NIH Policy Manual 3040–2, “Animal Care and Use in the Intramural Program” (PM 3040–2) and the NIH Animal Research Advisory Committee Guidelines (ARAC Guidelines). Those documents are posted on the NIH Office of Animal Care and Use public website at: http://oacu.od.nih.gov.

### qRT-PCR of viral RNA

Lungs and nasal turbinates collected for viral titration were stored at -80°C until processing. Tissues were weighed and homogenized in L15 media containing 1% antibiotic-antimycotic (Invitrogen) to a final 10% wt/vol. Homogenates were centrifuged for 10 minutes at 1500 rpm with a swinging bucket rotor (Sorvall 75006445). Viral RNA was then isolated from the homogenates using the QIAmp viral RNA mini kit (Qiagen) following manufacturer’s instructions. qRT-PCR reactions were amplified using 200ng of RNA per reaction with primer sets designed to detect MERS-CoV via the viral envelope (UpE) or nucleocapsid (N2 and N3) protein with the SuperScript III Platinum One-Step qRT-PCR kit (Life Technologies) [30]. Results are displayed using N3 primers, the confirmatory primer set. A sample from a naïve rabbit was always run to verify no background was detected with the N3 primer set. A standard dilution set of a titered virus stock was run in parallel, and all samples were tested in duplicate. Titers are expressed as log_10_ TCID_50_ equivalents per gram of tissue.

### Histopathology

Lung tissue samples from all lobes were resected from formalin-fixed tissue. Tissue was embedded in paraffin, sectioned at 5-μm, and stained with hematoxylin and eosin (Histoserv, Germantown, Maryland). Sections were examined by light microscopy (LM) or fluorescence microscopy (FM), using an Olympus BX51 microscope, and photomicrographs were taken using an Olympus DP73 (LM) camera or DP80 camera (FM). All histopathology scoring of tissues was blinded.

### Immunohistochemistry and Immunofluorescence

Lung sections were baked at 60°C for 1 hour then paraffin was removed with xylene and the sample was rehydrated with alcohol-gradated washes. Sections were microwaved with Antigen Unmasking Solution (Vector Laboratories), and then exposed to protein block (Dako) for 30 minutes. For immunohistochemistry (IHC) mouse anti-MERS nucleocapsid protein (NP) antibody (Biorbyt) was added at a dilution of 1:100, followed by biotinylated horse anti-mouse immunoglobulin G (IgG; Vector Laboratories) at a dilution of 1:200. Rat anti-CD3 (AbD Serotec; 1:100 dilution) and goat anti-DPP4/CD26 (R&D Systems; 1:25 dilution) antibodies were followed by a hydrogen peroxide blocking step for endogenous peroxidase activity, and then respective biotinylated goat anti-rat and horse anti-goat IgG antibodies (Vector Laboratories) at dilutions of 1:200. Detection of MERS NP was completed with incubations of 30 minutes with Vectastain ABC-AP reagent (Vector Laboratories) and 25 minutes with Vulcan Fast Red (Biocare). Detection of CD3 and DPP4 was completed with incubations of 30 minutes with Vectastain ABC RTU (Vector Laboratories) and 7.5 minutes with DAB. Immunofluorescence Antibody Assay (IFA) differentiated after the primary antibody incubations. Complement C9 antibody (MyBioSource) was added at a dilution of 1:50, followed by goat anti-guinea pig IgG (Vector Laboratories) at 1:200 and streptavidin conjugated to AlexaFluor 594 (Life Technologies) at 1:500. MERS NP (same as IHC) was detected with goat anti-mouse directly conjugated to AlexaFluor 488 at a 1:500 dilution. Slides were counterstained with hematoxylin (IHC) or DAPI (IFA) and evaluated by a veterinary pathologist.

### Digital quantitative pathology

Image analysis was performed on MERS-CoV infected lung tissues to provide a quantitative analysis of the MERS virus antigen present in the lung and the associated inflammatory response. Following histological (Hematoxylin and Eosin; H&E) and immunohistochemical evaluation of the lung sections, tissue slides were digitized with a bright-field Leica Aperio AT2 slide scanner at 40x magnification (S3 and S4 Figs). The images were evaluated using a web-based digital pathology information management system (eslide manager) used for both digital slide viewing and image analysis. The Aperio Positive Pixel Count (PPC) and Color Deconvolution V9 based algorithms were adjusted to recognize the inflammatory regions and the intensity of Vulcan Fast Red chromogen (Biocare Medical), respectively. In the previously stained and scanned lung sections, analysis was based on the settings of Hue Value and Hue Width (PPC) or values of the red, blue, and green channels (CD); gating and selection of regions of interest prevented the incorporation of nonspecific staining in the results. After these macros were optimized to suit the desired application, the settings were saved and were used for the evaluation of all the slides. The channel parameters for the H&E and MERS-specific macros were as follows: MERS-CoV H&E (PPC): Hue Value 0.647 and Hue Width 0.347 and MERS-CoV Fast Red (CD): red component 0.561, green component 0.679, and blue component 0.185.

### Microneutralization assay

Neutralizing activity in rabbit sera were evaluated by a microneutralization (MN) assay. To determine the antibody titers, serial two-fold dilutions of sera were prepared. 100 TCID_50_ of virus was mixed with the sera in equal volume and incubated for one hour at room temperature, before the mixture was subsequently added in quadruplicate to Vero81 cell monolayer. The serum neutralization titer was determined as the reciprocal of the serum dilution that neutralized virus as evidenced by the absence of any cytopathic effect on day 4 and confirmed on day 6.

### MERS-CoV Spike Protein Anti-IgG Capture ELISA

To quantify anti-S protein IgG antibodies from rabbit serum, 96-well plates were coated overnight with 100ng/well of recombinant MERS-CoV S protein (Sino Biological) in sodium bicarbonate buffer. Subsequently, the plates were blocked for 2 hours at room temperature with 10% FBS in PBS. Plates were washed and incubated for 2 hours with serial four-fold dilutions of heat-inactivated rabbit serum in duplicate. The plates were washed and further incubated at room temp with HRP conjugated goat anti-rabbit IgG (Abcam ab6721) diluted 1:120,000 in PBS with 5% BSA and 0.05% Tween-20. For detection, following additional washes, SureBlue TMB Microwell Peroxidase Substrate (KPL) was added to each well and TMB BlueSTOP solution (KPL) was added after 10 minutes. The optical density of each well was measured at 650 nm on a SpectraMax i3 plate reader (Molecular Devices) and an OD greater than two standard deviations above the mean of the background was considered positive.

### MERS-CoV Nucleocapsid Protein ELISA

To examine anti-N protein IgG antibodies from rabbit serum, we utilized an ELISA protocol developed by the CDC [61]. Briefly, 96-well plates were coated overnight with purified MERS-CoV N antigen or irrelevant control antigen (both obtained from Division of Viral Diseases, Centers for Disease Control and Prevention) in PBS. Plates were then washed and serial four-fold dilutions of heat-inactivated rabbit serum were added for one hour at 37°C. After incubation the plates were washed further and incubated with HRP conjugated goat anti-rabbit IgG (Abcam ab6721) diluted 1:120,000 in PBS with 5% BSA and 0.05% Tween-20 for one hour at 37°C. Following additional washes, positive sera were determined by the addition of ABTS Peroxidase substrate solution (KPL) that was incubated for 30 minutes at 37°C, followed by the addition of ABTS stop solution (KPL). The optical density of each well was measured at 405 nm on a SpectraMax i3 plate reader (Molecular Devices) and an OD of 0.3 above the negative control was considered positive.

### Rabbit C3a ELISA

To compare the amounts of C3a present in rabbit lungs following infection with MERS-CoV, we utilized the Rabbit Complement Fragment 3a (C3a) ELISA kit (MBS703171, MyBioSource), according to manufacturer’s instructions. Frozen rabbit lung samples were rinsed and homogenized to 10% w/v in PBS, and stored overnight at -20°C. Following two freeze-thaw cycles, the samples were centrifuges at 5000g for 5 minutes and the supernatants were assayed immediately, in triplicate. Undiluted samples were added to pre-coated plates for 2hrs at 37°C. The samples were removed, and the biotin-antibody was added for 1hr at 37°C. The plates were then washed 3 times before the addition of the HRP-avidin antibody for 1hr at 37°C. The plates were washed 5 times, before incubation with the TMB Substrate for 20min at 37°C. Stop solution was then added, and the OD was measured at 450nm within 5 minutes. Samples were quantitated based on a standard dilution series within the plate.

### Antibody-dependent enhancement assay

To determine if antibodies resulted in increased viral replication in macrophages, we conducted an antibody-dependent enhancement (ADE) assay. THP-1 cells were differentiated into macrophages by addition of 20nM PMA into the RPMI media for 24 hours, followed by a week of culturing without PMA. The cells became adherent to the flask, and took on a macrophage-like appearance. The differentiation of THP-1 cells was confirmed by immunofluorescence with the loss of CD14 and increase of CD36, CD68, and CD71 on the cell surface compared to undifferentiated THP-1 cells, adapted from Genin et al [62]. For a positive control, we infected Vero81 cells. As a negative control, we included Raji cells, which lack both DPP4 and Fc receptors. Heat-inactivated rabbit sera at three dilutions (undiluted, 1:10, and 1:100) were incubated with EMC/2012 at an MOI of 1 for 1 hour at 37°C before addition onto each cell type in duplicate in 96-well plates for 2 hours at 37°C. Cells were then washed and incubated for 48 hours before supernatants were collected for viral titration.

### Statistical analysis

Mean viral titers are displayed with the standard error of the mean. Statistical significance was determined using one-way ANOVA with multiple comparisons tests in GraphPad Prism v7.

## Supporting information

S1 Fig Virus titers in the respiratory tract following primary infection with MERS-CoV. Virus titers in the nasal turbinates (A) and lungs (B) of rabbits following infection with either 10^3^ or 10^5^ TCID_50_ of EMC/2012 strain of MERS-CoV through day 5 after infection, as determined by titration in Vero81 cells.
(TIFF)

S2 Fig Virus titers in the upper respiratory tract following reinfection with MERS-CoV. Viral RNA titers in the nasal turbinates of rabbits following primary infection or reinfection with EMC/2012. n = 3 rabbits per group.
(TIFF)

S3 Fig Digital quantification of viral antigen. Images are shown for H&E (top), IHC (middle), and Color Deconvolution Algorithm (CDA)(bottom). Images show the entire lung section that was analyzed (A), an area magnified to 10x (B), and to 20x (C) for clarity. Dashed boxes indicate regions of interest, and BV are blood vessels for orientation. On the CDA images, red indicates areas of the most intense (concentrated) viral antigen deposition, yellow indicates areas of less intense (moderate) viral antigen deposition, and purple areas are virus antigen negative.
(TIFF)

S4 Fig Digital quantification of inflammation in rabbit lung lobes. Images are shown for H&E (left) and Positive Pixel Count (PPC) Algorithm (right). Images show the entire lung sections that were analyzed for the presence inflammatory areas (top), with an area magnified to 4x (bottom). Examples are shown of a lung lobe with abundant inflammation (A, B) and where inflammation was minimal to absent (C, D). On the algorithm images, red indicates areas positive for (inflammatory) cells and blue areas represent regions that are negative for inflammation. Positivity (% of lung lobe positive for inflammatory cell nuclei) is measured by the number of positive cells over the total number of cells in the lobe.
(TIFF)

S1 Table Qualitative IHC and histopathology scoring of lungs from MERS-CoV infected rabbits. (DOCX)

S2 Table Digital quantitative IHC scoring of viral antigen in lungs from MERS-CoV infected rabbits. (DOCX)

S3 Table Digital quantitative H&E scoring of inflammation by counting inflammatory cell nuclei in lung sections from subset of MERS-CoV infected rabbits. (DOCX)
