# Supplementary figures and images for: Enhanced inflammation in New Zealand white rabbits when MERS-CoV reinfection occurs in the absence of neutralizing antibody
Source: PLoS Pathog. 2017 Aug 17;13(8):e1006565. doi: 10.1371/journal.ppat.1006565 (PMC5574614; doi:10.1371/journal.ppat.1006565)

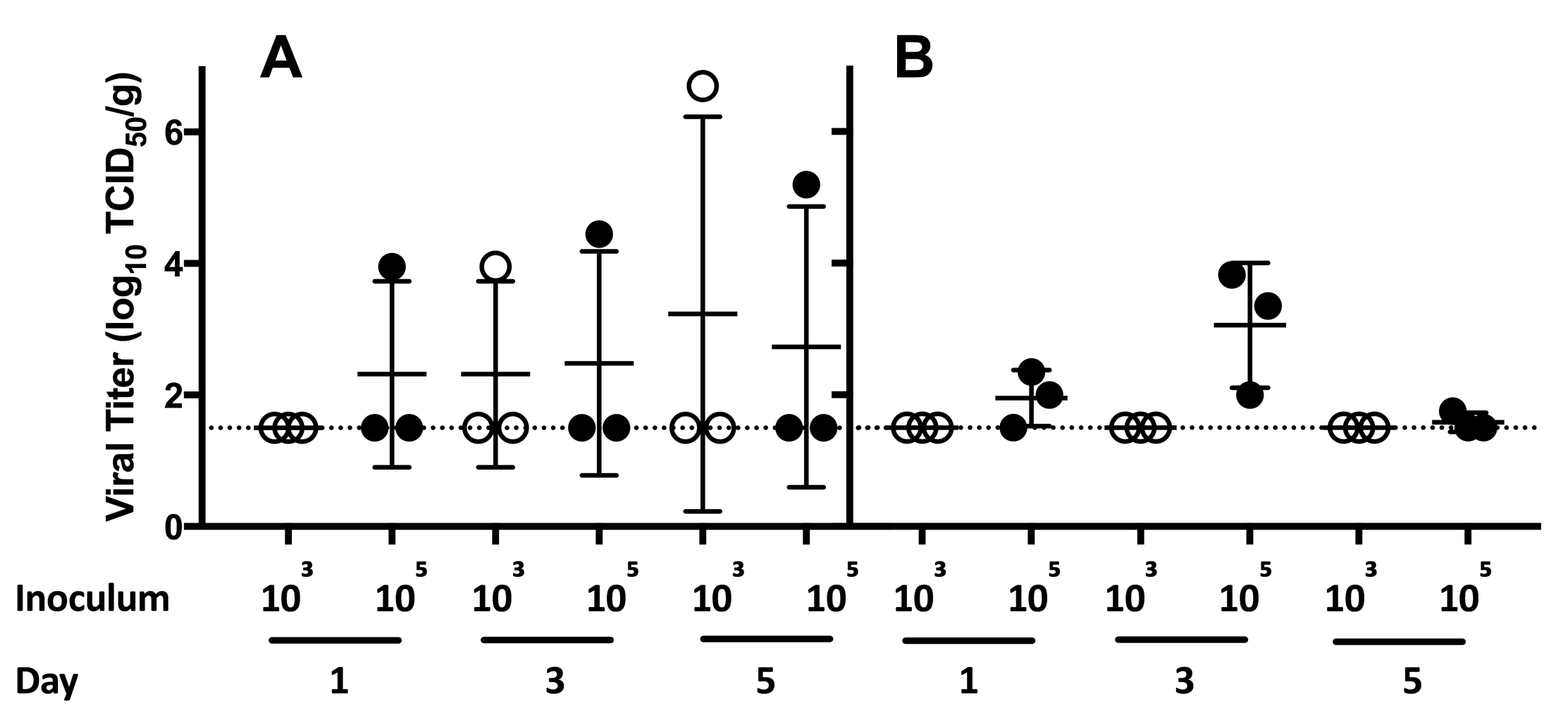

Supplement: S1 Fig — Virus titers in the nasal turbinates (A) and lungs (B) of rabbits following infection with either 103 or 105 TCID50 of EMC/2012 strain of MERS-CoV through day 5 after infection, as determined by titration in Vero81 cells. (TIFF) [file ppat.1006565.s001.tiff]

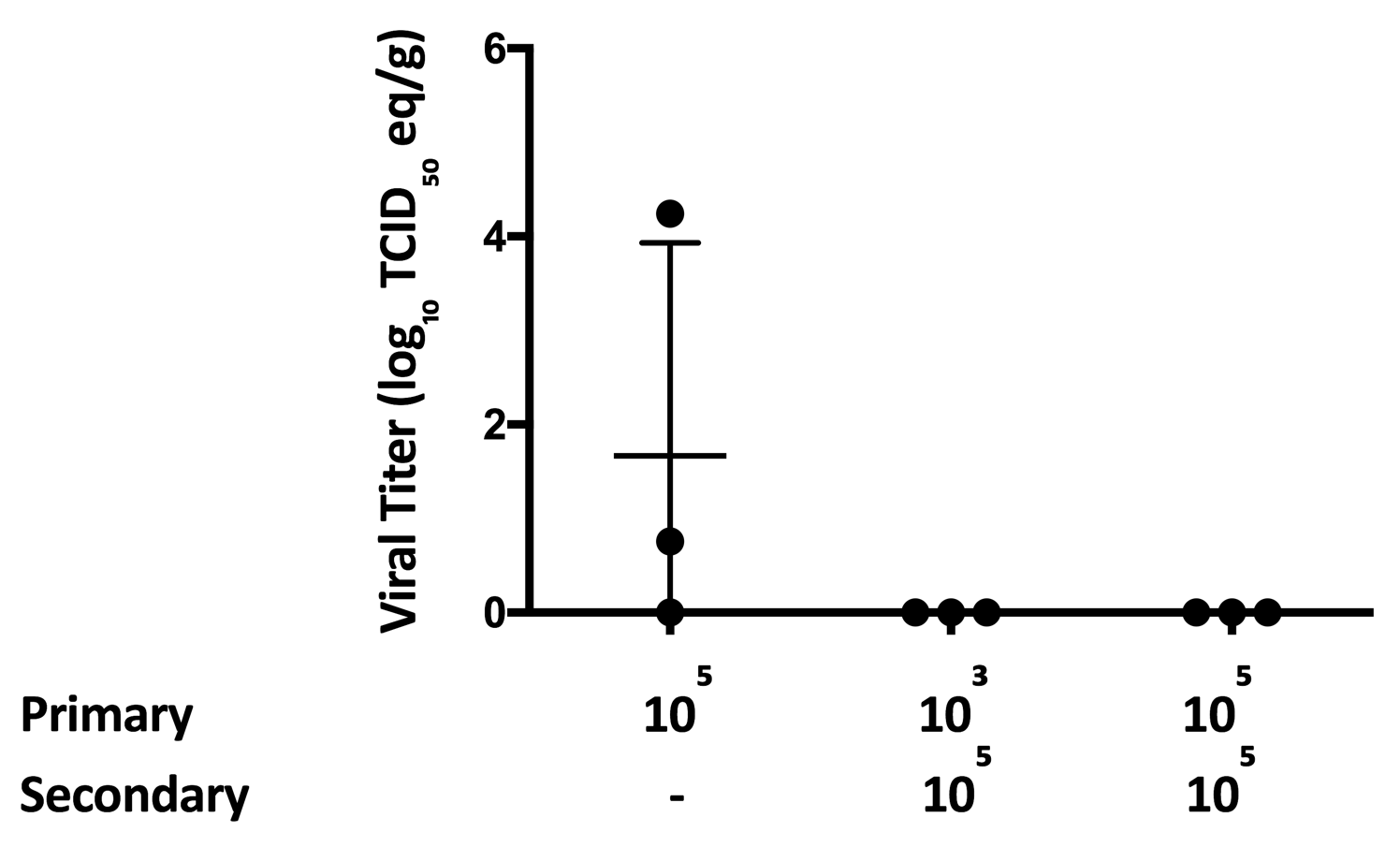

Supplement: S2 Fig — Viral RNA titers in the nasal turbinates of rabbits following primary infection or reinfection with EMC/2012. n = 3 rabbits per group. (TIFF) [file ppat.1006565.s002.tiff]

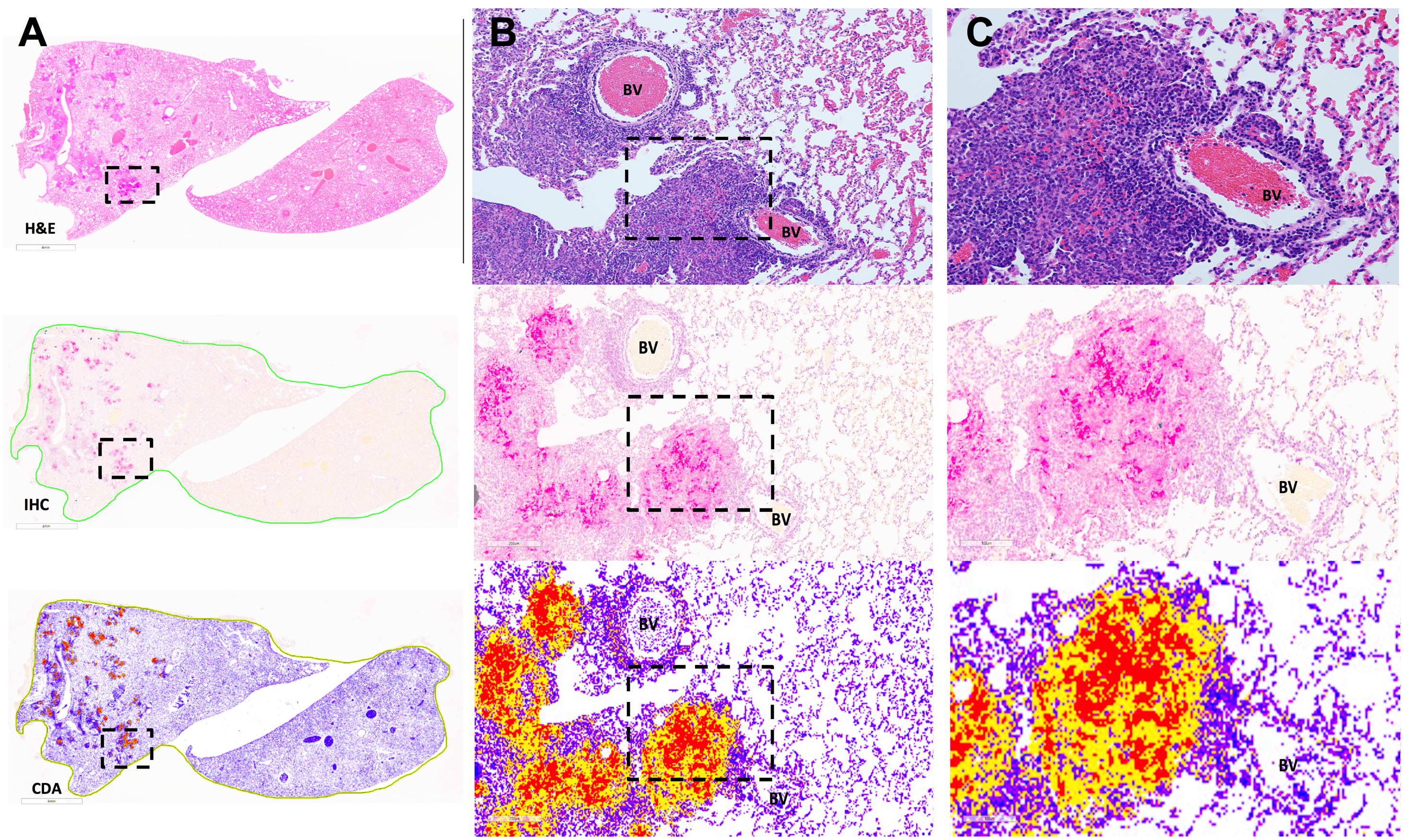

Supplement: S3 Fig — Images are shown for H&E (top), IHC (middle), and Color Deconvolution Algorithm (CDA)(bottom). Images show the entire lung section that was analyzed (A), an area magnified to 10x (B), and to 20x (C) for clarity. Dashed boxes indicate regions of interest, and BV are blood vessels for orientation. On the CDA images, red indicates areas of the most intense (concentrated) viral antigen deposition, yellow indicates areas of less intense (moderate) viral antigen deposition, and purple areas are virus antigen negative. (TIFF) [file ppat.1006565.s003.tiff]

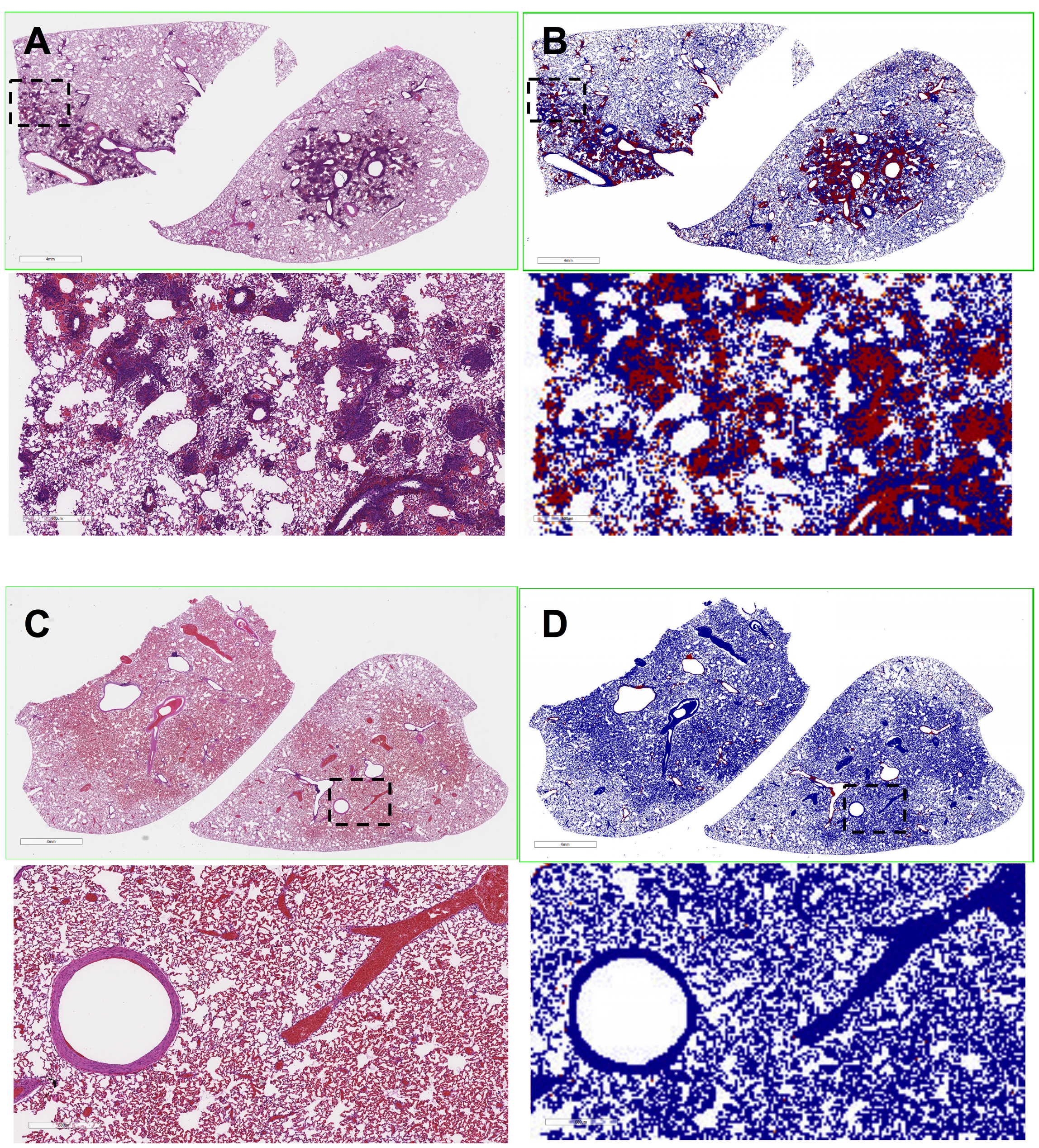

Supplement: S4 Fig — Images are shown for H&E (left) and Positive Pixel Count (PPC) Algorithm (right). Images show the entire lung sections that were analyzed for the presence inflammatory areas (top), with an area magnified to 4x (bottom). Examples are shown of a lung lobe with abundant inflammation (A, B) and where inflammation was minimal to absent (C, D). On the algorithm images, red indicates areas positive for (inflammatory) cells and blue areas represent regions that are negative for inflammation. Positivity (% of lung lobe positive for inflammatory cell nuclei) is measured by the number of positive cells over the total number of cells in the lobe. (TIFF) [file ppat.1006565.s004.tiff]
