# Supplementary material for: Enhanced inflammation in New Zealand white rabbits when MERS-CoV reinfection occurs in the absence of neutralizing antibody
Source: PLoS Pathog. 2017 Aug 17;13(8):e1006565. doi: 10.1371/journal.ppat.1006565 (PMC5574614; doi:10.1371/journal.ppat.1006565)
Supplement: S1 Table — (DOCX) [file ppat.1006565.s005.docx]

S1 Table. Qualitative IHC and histopathology scoring of lungs from MERS-CoV infected rabbits.

|  |  |  | IHC- Viral Antigen | Histopathology |
| --- | --- | --- | --- | --- |
| Experiment | Virus Dose(s) TCID_50_/ml^a^ | Day Post-Infection^b^ | Score per lobe^c^ (LCd/RCd/LCr/RCr) | Score per lobe (LCd/RCd/LCr/RCr) |
| Primary Infection- EMC | 10^3^ | 1 | 0/0/0/0 | 0/0/0/0 |
|  |  |  | 0/0/0/0 | 0/0/0/0 |
|  |  |  | 0/0/0/0 | 0/0/0/0 |
|  |  | 3 | 0/0/0/0 | 0/0/0/0 |
|  |  |  | 0/0/0/0 | 0/0/0/0 |
|  |  |  | 0/0/0/0 | 0/0/0/0 |
|  |  | 5 | 0/0/0/0 | 0/0/0/0 |
|  |  |  | 0/0/0/0 | 0/0/0/0 |
|  |  |  | 0/0/0/0 | 0/0/0/0 |
|  | 10^5^ | 1 | 0/1/0/1 | 0/0/0/0 |
|  |  |  | 0/0/1/0 | 0/0/0/0 |
|  |  |  | 1/0/0/1 | 0/0/0/0 |
|  |  | 3 | 1/0/0/0 | 0/1/1/3 |
|  |  |  | 0/0/4/0 | 0/2/1/0 |
|  |  |  | 0/0/3/0 | 0/0/0/0 |
|  |  | 5 | 0/2/0/0 | 0/1/0/0 |
|  |  |  | 0/2/0/0 | 0/0/0/0 |
|  |  |  | 0/2/0/0 | 0/1/1/1 |
| Secondary Infection- EMC | 10^3//^10^5^ | 3 | 4/3/0/0 | 3/2/0/0 |
|  |  |  | 0/4/1/3 | 0/3/1/1 |
|  |  |  | 0/1/2/3 | 0/0/1/1 |
|  | 10^5//^10^5^ | 3 | 0/0/0/0 | 0/0/0/0 |
|  |  |  | 0/3/0/0 | 0/2/0/0 |
|  |  |  | 1/4/4/3 | 0/2/3/2 |
|  | None^//^10^5^ | 3 | 3/4/1/1 | 2/3/1/0 |
|  | (Primary control) |  | 1/0/3/0 | 0/0/2/0 |
|  |  |  | 1/4/0/2 | 0/3/0/0 |
|  | 10^3//^media | 3 | 0/0/0/0 | 0/0/0/0 |
|  |  |  | 0/0/0/0 | 0/0/0/0 |
|  |  |  | 0/0/0/0 | 0/0/0/0 |
|  | 10^5//^media | 3 | 0/0/0/0 | 0/0/0/0 |
|  |  |  | 0/0/0/0 | 0/0/0/0 |
|  |  |  | 0/0/0/0 | 0/0/0/0 |
|  | media^//^media |  | 0/0/0/0 | 0/0/0/0 |
|  |  |  | 0/0/0/0 | 0/0/0/0 |
|  |  |  | 0/0/0/0 | 0/0/0/0 |
| Tertiary Infection- EMC | 10^3//^10^5//^10^5^ | 3 | 0/3/2/0 | 0/2/1/0 |
|  |  |  | 0/0/0/0 | 0/0/0/0 |
|  |  |  | 0/0/3/0 | 2/1/3/0 |
|  | 10^5//^10^5//^10^5^ | 3 | 0/1/0/1 | 2/3/1/0 |
|  |  |  | 0/0/0/1 | 1/1/1/3 |
|  |  |  | 0/0/0/0 | 0/0/0/0 |
|  | None^//^None^//^10^5^ | 3 | 3/0/2/2 | 0/0/1/1 |
|  | (Primary control) |  | 1/0/2/2 | 0/0/1/1 |
|  |  |  | 2/1/2/0 | 1/0/1/0 |
| Passive Transfer (PT)- EMC | 10^3//^10^5^ | 3 | 4/0/0/1 | 1/0/0/0 |
|  | No PT |  | 2/0/0/0 | 1/0/0/0 |
|  |  |  | 4/0/3/1 | 2/0/1/0 |
|  | 10^3//^10^5^ | 3 | 0/2/0/2 | 0/1/0/0 |
|  | PT |  | 3/0/1/0 | 0/0/2/0 |
|  |  |  | 2/0/1/0 | 1/1/1/0 |
|  | 10^3//^10^5^ | 3 | 2/2/0/0 | 0/0/1/0 |
|  | 1:10 PT |  | 0/1/0/2 | 0/0/0/0 |
|  |  |  | 0/2/0/0 | 0/0/0/0 |

^a^ ^//^ indicates the sequence of subsequent infections

^b^ The day listed is relative to day of intranasal infection with MERS-CoV.

^c^ The scoring of IHC and histopathology identifies each lobe in the following order: Left Caudal, Right Caudal, Left Cranial, Right Cranial. The IHC scores indicate the following antigen distribution per section: 0- no antigen, 1- less than 5%, 2- between 5 and 20%, 3- between 21 and 50%, 4- between 51 and 75%, and 4- more than 75%. The histopathology score indicate the following severity per section: 0- less than 10%, 1- between 10 to 30%, 2- between 31 and 50%, 3- between 51 and 75%, 4- between 76 and 90%, and 5- more than 90%.

None- No inoculation was performed at the indicated timepoint.
