# Supplementary material for: Enhanced inflammation in New Zealand white rabbits when MERS-CoV reinfection occurs in the absence of neutralizing antibody
Source: PLoS Pathog. 2017 Aug 17;13(8):e1006565. doi: 10.1371/journal.ppat.1006565 (PMC5574614; doi:10.1371/journal.ppat.1006565)
Supplement: S2 Table — (DOCX) [file ppat.1006565.s006.docx]

S2 Table. Digital quantitative IHC scoring of viral antigen in lungs from MERS-CoV infected rabbits.

|  |  |  | Left caudal lobe | | Right caudal lobe | | Number of rabbits positive for antigen/total rabbits^c^ |
| --- | --- | --- | --- | --- | --- | --- | --- |
| Experiment | Virus Dose(s) TCID_50_/ml^a^ | Day Post-Infection^b^ | % strong positive | % medium positive | % strong positive | % medium positive |  |
| Primary Infection- EMC | 10^3^ | 1 | 0.0 | 0.0 | 0.0 | 0.0 | 0/3 |
|  |  |  | 0.0 | 0.0 | 0.0 | 0.0 |  |
|  |  |  | 0.0 | 0.0 | 0.0 | 0.0 |  |
|  |  | 3 | 0.0 | 0.1 | 0.0 | 0.1 | 0/3 |
|  |  |  | 0.0 | 0.1 | 0.0 | 0.1 |  |
|  |  |  | 0.0 | 0.1 | 0.0 | 0.0 |  |
|  |  | 5 | 0.0 | 0.0 | 0.0 | 0.4 | 0/3 |
|  |  |  | 0.0 | 0.0 | 0.0 | 0.0 |  |
|  |  |  | 0.0 | 0.0 | 0.0 | 0.1 |  |
|  | 10^5^ | 1 | 0.0 | 0.0 | 0.0 | 0.0 | 0/3 |
|  |  |  | 0.0 | 0.0 | 0.0 | 0.0 |  |
|  |  |  | 0.0 | 0.5 | 0.0 | 0.1 |  |
|  |  | 3 | 0.0 | 0.4 | 0.4 | **1.7^d^** | 3/3 |
|  |  |  | 0.0 | 0.0 | 0.0 | **1.3** |  |
|  |  |  | 0.0 | 0.1 | 0.7 | **6.4** |  |
|  |  | 5 | 0.0 | 0.0 | 0.2 | **3.3** | 3/3 |
|  |  |  | 0.0 | 0.0 | 0.3 | **3.5** |  |
|  |  |  | 0.0 | 0.0 | 0.0 | **1.4** |  |
| Secondary Infection- EMC | 10^3//^10^5^ | 3 | **5.6** | **15.8** | **3.6** | **10.2** | 3/3 |
|  |  |  | 0.0 | 0.0 | **7.0** | **20.0** |  |
|  |  |  | 0.0 | 0.0 | **1.7** | **5.2** |  |
|  | 10^5//^10^5^ | 3 | 0.0 | 0.0 | 0.0 | 0.1 | 2/3 |
|  |  |  | 0.0 | 0.0 | **1.8** | **5.0** |  |
|  |  |  | **1.0** | **4.4** | **4.7** | **9.0** |  |
|  | None^//^10^5^ | 3 | 0.7 | **1.7** | **6.1** | **21.9** | 2/3 |
|  | (Primary control) |  | 0.0 | 0.2 | 0.0 | 0.1 |  |
|  |  |  | 0.4 | **1.1** | **4.8** | **14.5** |  |
|  | 10^3//^media | 3 | 0.0 | 0.0 | 0.0 | 0.0 | 0/3 |
|  |  |  | 0.0 | 0.0 | 0.0 | 0.1 |  |
|  |  |  | 0.0 | 0.0 | 0.0 | 0.2 |  |
|  | 10^5//^media | 3 | 0.0 | 0.0 | 0.0 | 0.1 | 0/3 |
|  |  |  | 0.0 | 0.0 | 0.0 | 0.1 |  |
|  |  |  | 0.0 | 0.0 | 0.0 | 0.1 |  |
|  | media^//^media |  | 0.0 | 0.0 | 0.0 | 0.0 | 0/3 |
|  |  |  | 0.0 | 0.0 | 0.0 | 0.0 |  |
|  |  |  | 0.0 | 0.0 | 0.0 | 0.1 |  |
| Tertiary Infection- EMC | 10^3//^10^5//^10^5^ | 3 | 0.0 | 0.0 | 0.2 | **1.1** | 1/3 |
|  |  |  | 0.0 | 0.0 | 0.0 | 0.0 |  |
|  |  |  | 0.0 | 0.0 | 0.0 | 0.1 |  |
|  | 10^5//^10^5//^10^5^ | 3 | 0.0 | 0.0 | 0.0 | 0.1 | 0/3 |
|  |  |  | 0.0 | 0.0 | 0.0 | 0.1 |  |
|  |  |  | 0.0 | 0.0 | 0.0 | 0.1 |  |
|  | None^//^ None^//^10^5^ | 3 | **1.4** | **3.7** | 0.0 | 0.2 | 2/3 |
|  | (Primary control) |  | 0.0 | 0.2 | 0.0 | **1.0** |  |
|  |  |  | 0.1 | 0.7 | 0.1 | 0.7 |  |
| Passive Transfer (PT)- EMC | 10^3//^10^5^ | 3 | **1.0** | **4.8** | 0.0 | 0.0 | 3/3 |
|  | No PT |  | 0.1 | **1.2** | 0.0 | 0.0 |  |
|  |  |  | **1.7** | **9.2** | 0.0 | 0.0 |  |
|  | 10^3//^10^5^ | 3 | 0.0 | 0.0 | 0.3 | 0.9 | 1/3 |
|  | PT |  | 0.9 | **1.8** | 0.0 | 0.0 |  |
|  |  |  | 0.2 | 0.7 | 0.0 | 0.0 |  |
|  | 10^3//^10^5^ | 3 | 0.1 | 0.7 | 0.0 | 0.0 | 0/3 |
|  | 1:10 PT |  | 0.0 | 0.0 | 0.0 | 0.0 |  |
|  |  |  | 0.0 | 0.0 | 0.0 | 0.0 |  |

^a^ ^//^ indicates the sequence of subsequent infections

^b^ The day listed is relative to day of intranasal infection with MERS-CoV.

^c^ Combined percent of medium and strong positivity in the sample must total ≥ 1% in a single lobe in order to be considered positive for viral antigen. Weak positivity was not considered.

^d^ Values of positivity for either medium or strong staining are bolded when ≥ 1%.

None- No inoculation was performed at the indicated timepoint.
