# Supplementary material for: Enhanced inflammation in New Zealand white rabbits when MERS-CoV reinfection occurs in the absence of neutralizing antibody
Source: PLoS Pathog. 2017 Aug 17;13(8):e1006565. doi: 10.1371/journal.ppat.1006565 (PMC5574614; doi:10.1371/journal.ppat.1006565)
Supplement: S3 Table — (DOCX) [file ppat.1006565.s007.docx]

S3 Table. Digital quantitative H&E scoring of viral antigen in lungs from subset of MERS-CoV infected rabbits.

|  |  |  | % of lung lobe positive for inflammatory cell nuclei^b^ | |
| --- | --- | --- | --- | --- |
| Experiment | Virus Dose(s) TCID_50_/ml^a^ | Day Post-Infection^b^ | Left caudal lobe | Right caudal lobe |
| Primary Infection- EMC | 10^3^ | 3 | 3.4 | 13.6 |
|  |  |  | 3.2 | 11.1 |
|  |  |  | 7.5 | 9.5 |
|  | 10^5^ | 3 | 0.4 | 2.2 |
|  |  |  | 4.1 | **22.1** |
|  |  |  | 0.2 | 2.7 |
| Secondary Infection- EMC | 10^3//^10^5^ | 3 | 5.8 | 13.6 |
|  |  |  | **16.1^c^** | **25.5** |
|  |  |  | 4.3 | **28.3** |
|  | 10^5//^10^5^ | 3 | 2.5 | 14.0 |
|  |  |  | 4.0 | **19.9** |
|  |  |  | 1.1 | **24.1** |
|  | None^//^10^5^ | 3 | 0.9 | 4.9 |
|  | (Primary control) |  | 1.1 | 2.3 |
|  |  |  | 14.5 | **16.9** |
| Tertiary Infection- EMC | 10^5//^10^5//^10^5^ | 3 | 7.1 | **18.5** |
|  |  |  | 0.4 | 1.1 |
|  |  |  | 0.4 | 3.9 |

^a^ ^//^ indicates the sequence of subsequent infections

^b^ Baseline average value of inflammatory cells in naïve rabbit lungs were determined to be 7.2%. All values therefore have a standard deviation of ± 7%.

^c^ Values of positivity are bolded when ≥ 15% (two times the standard deviation).

None- No inoculation was performed at the indicated time point.
